# Environmental evolution, faunal and human occupation since 2 Ma in the Anagni basin, central Italy

**DOI:** 10.1038/s41598-021-85446-5

**Published:** 2021-03-29

**Authors:** Fabio Florindo, Fabrizio Marra, Diego E. Angelucci, Italo Biddittu, Luciano Bruni, Federico Florindo, Mario Gaeta, Hervé Guillou, Brian Jicha, Patrizia Macrì, Caterina Morigi, Sebastien Nomade, Fabio Parenti, Alison Pereira, Stefano Grimaldi

**Affiliations:** 1 Istituto Nazionale di Geofisica e Vulcanologia, Rome, Italy; 2 Institute for Climate Change Solutions, Via Sorchio snc, 61040 Frontone, Italy; 3 Department of Humanities, University of Trento, Trento, Italy; 4 Istituto Italiano di Paleontologia Umana, Anagni, Italy; 5 Sapienza Università di Roma, Piazzale Aldo Moro 5, 00185 Roma, Italy; 6 Dipartimento di Scienze della Terra, Sapienza Università di Roma, Piazzale Aldo Moro 5, 00185 Rome, Italy; 7 LSCE/IPSL, UMR CEA-CNRS-UVSQ 8212, Laboratoire des Sciences du Climat et de l’Environnement, CEA Saclay, Bat 714, Chemin de Saint Aubin - RD 128, 91191 Gif sur Yvette, France; 8 Department of Geoscience, University of Wisconsin-Madison, Madison, USA; 9 Department of Earth Sciences, University of Pisa, Via S. Maria 53, 56126 Pisa, Italy; 10 Universidade Federal do Paraná, Curitiba, Brazil; 11 CNRS Laboratoire GEOPS, Université Paris-Saclay, Orsay, France; 12 Département Hommes et Environnements, Muséum national d’Histoire naturelle, Paris, France

**Keywords:** Stratigraphy, Geology, Palaeomagnetism, Archaeology

## Abstract

We present the study of a composite, yet continuous sedimentary succession covering the time interval spanning 2.6–0.36 Ma in the intramontane basin of Anagni (central Italy) through a dedicated borecore, field surveys, and the review of previous data at the three palaeontological and archaeological sites of Colle Marino, Coste San Giacomo and Fontana Ranuccio. By combining the magneto- and chronostratigraphic data with sedimentologic and biostratigraphic analysis, we describe the palaeogeographic and tectonic evolution of this region during this entire interval. In this time frame, starting from 0.8 Ma, the progressive shallowing and temporary emersion of the large lacustrine basins and alluvial plains created favorable conditions for early hominin occupation of the area, as attested by abundant tool industry occurrences and fossils. This study provides new constraints to better interpret the hominin migratory dynamics and the factors that influenced the location and spatial distribution during the early occupation of this region.

## Introduction

**Figure 1:**
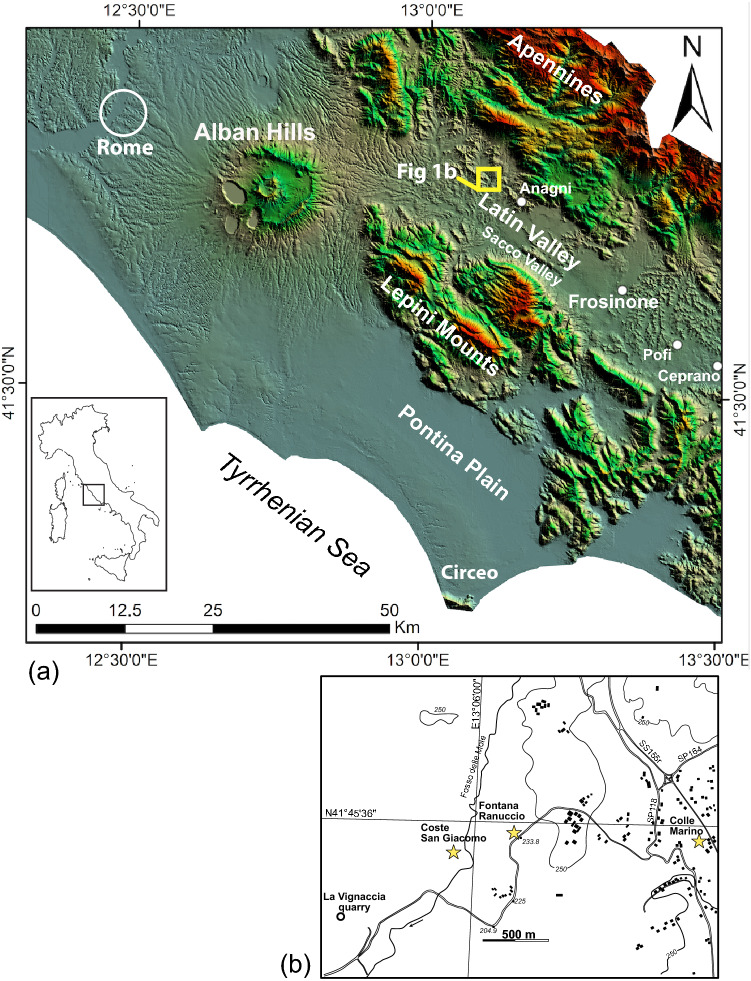
(**a**) Digital Elevation Model (DEM) image TINITALY/01 square WA 6570, of central Italy showing location of the Latin Valley and (**b**) of the investigated archaeological sites. Used with permission of the Istituto Nazionale di Geofisica e Vulcanologia, Rome.

Since the late 1960s, scholars of the Italian Institute of Human Palaeontology (hereafter, the IIPU) have carried out field surveys, archaeological excavations, and geomorphological studies in the Latin Valley, Central Italy (Fig. 1). This area is renowned for the abundant palaeontological, archaeological, and palaeoanthropological occurrences, including the finding in 1994 of a calvarium at the Ceprano locality, initially considered as the oldest evidence of human presence in the Italian peninsula^1–3^. Despite the large number of reports, few chronologic constraints have been achieved for the faunal and lithic assemblages recovered at several sites in the Latin Valley until recently. The first integrated, modern approach to the study of these sites was performed at Fontana Ranuccio (FR) and at Coste San Giacomo (CSG), where an outstanding vertebrate faunal record of Villafranchian age was obtained^4–10^.

Through combined sedimentological, palaeomagnetic, palynological and palaeontological investigations, an age of ca. 2 Ma for CSG was suggested and the site was proposed as the type-locality for the homonymous Faunal Unit of the Middle Villafranchian^4,5^. More recently, the application of the ^40^Ar/^39^Ar dating method on the volcanic deposits intercalated within the sedimentary successions of the Latin Valley has led to provide precise ages to several important Middle Pleistocene archaeological sites^11,12^. Among these, the abovementioned Ceprano site, as well as FR, where one of the richest bone-tool assemblages of Italy was recovered^6,10,13,14^, along with four human teeth^15–17^, lithic assemblage^18^, and a large vertebrate bone fossil record, which constitutes the local fauna for the homonymous, Late Galerian Faunal Unit of the Aurelian Mammal Age^19,20^.

Other localities in this region contain hominin remains attributed to *Homo heidelbergensis* such as those recovered during excavations at the Cava Pompi site of Pofi in 1961 and 1976 along with lithic and bone industries^21,22^. Similar assemblages also occur at Lademagne and Isoletta^6,23^.

A much younger age than previously though was determined for “Ceprano man” based on a 353 ± 4 ka date of an ash layer interbedded in the lacustrine deposits hosting the human calvarium^11^. An age of 408 ± 10 ka was suggested for the FR Acheulian lithic assemblage, revising previous estimations of ca. 458 ka^6^, along with similar ages around 400 ka for the Lademagne, Isoletta, and Cava Pompi sites by^12^.

In contrast to the great improvements achieved on the chronologic constraints of the archaeological sites mentioned above, no new investigations have been delivered at Colle Marino (CM) since its discovery^6^, despite the potential for groundbreaking implications of the findings reported at this site, which is located two kilometers east of CSG and FR. Indeed, a lithic assemblage—comprising uni- and bifacial choppers, polyhedrons, flakes, and pebble scrapers—was found on surface^6^, and its resemblance with the Oldowan (Mode I) industry, the oldest-known stone tool industry, was also suggested^24^. The potential archaeological layer of CM was correlated with a sedimentologically similar horizon, occurring at the same elevation, and immediately overlying the Middle Villafranchian fossiliferous layer of CSM, suggesting quite an ancient age (i.e., more than 0.7 Ma) for the lithic assemblage, consistent with the alleged Oldowan features^6^. Despite the presence of faunal remains of the ancient hyena *Pachycrocuta brevirostris* in the continental deposits of CM^10^, which correspond to a wide time interval between 1.8 and 0.8 Ma, the lithic assemblage recovered at CM was never re-investigated and its puzzling occurrence has been met with skepticism^25^ probably due to the lack of suitable chronologic markers.

To further understand the CM archaeological horizon and provide reliable age constraints for it, the IIPU and the Istituto Nazionale di Geofisica e Vulcanologia (hereafter, INGV) set up a research project in this area. In August 2020, a dedicated borehole was drilled at this locality in addition to field investigations and a systematic review of the log data from previous boreholes performed at the CSM and FR archaeological sites (Fig. 1).

The aim of the coring was to recover the sedimentary successions in the elevation range and correlate them with the one from CSG and with the archaeological layer outcropping at CM, to carry out litho-, bio-, and magneto-stratigraphic analyses, combined with ^40^Ar/^39^Ar dating of possible tephra layers, as well as of detrital sanidine (see^26^ for this methodology).

### Geological setting

**Figure 2:**
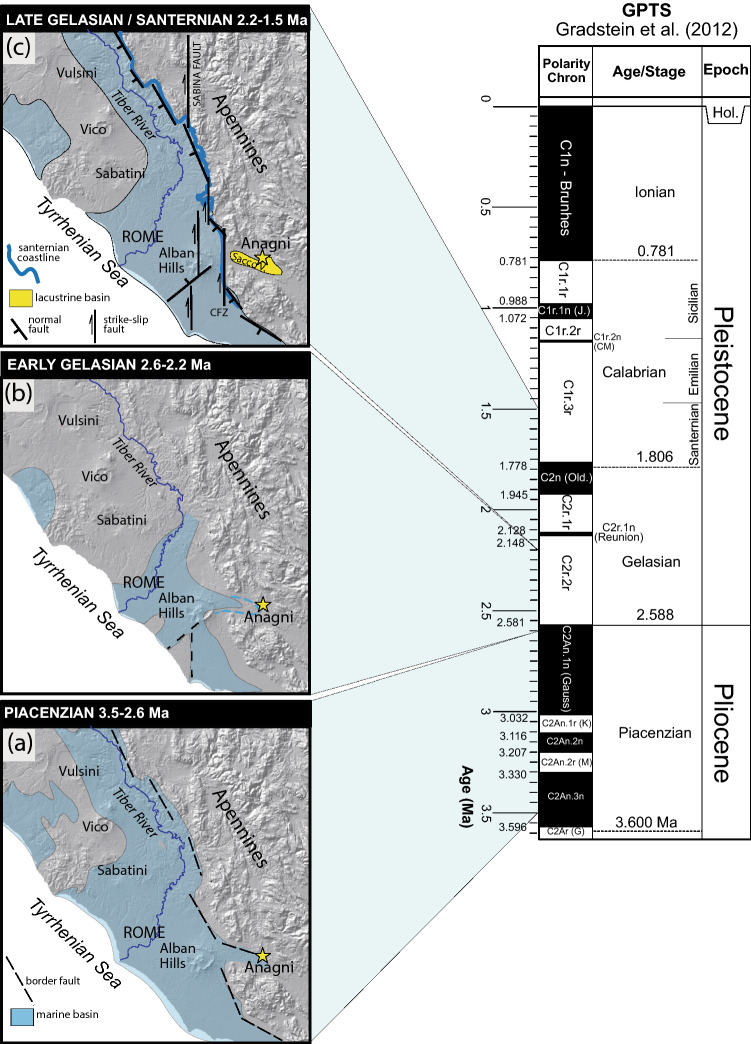
Palaeogeographic evolution of the Tyrrhenian Sea margin of central Italy according to^54^ and data from this work. GPTS = Geomagnetic Polarity Time Scale.

The foothills region of the Central Apennines is located between an arc-shaped fold-and-thrust belt and the Tyrrhenian Sea margin. The region hosts several palaeontological and archaeological sites which yielded a record of the faunal and early human occupation during the last two million years. The distribution of such occurrences is direct consequence of the palaeogeographic evolution of this region, a back-arc extensional domain (e.g.,^27,28^), which underwent to a progressive continentalization due to the uplift of the Apennine chain and to the southwest migration of the Tyrrhenian Sea basin and its coastline. Due to the temporal and spatial variations of the continental environments, the outcrops of sedimentary deposits featuring the oldest terrestrial faunal records, which are Middle to Late Villafranchian in age, are scattered within a narrow belt at the western foot of the Apennine range. This is an elongated area parallel to the coastline of the maximum marine ingression culminated during the Santernian (i.e., the local marine stage of the Lower Pleistocene spanning 1.8–1.5 Ma) (Fig. 2). The Latin Valley, a tectonic depression subjected to an extensional regime since the late Pliocene that led to the formation of lacustrine basins (e.g. Anagni and Ceprano), is located at the southern margin of this area^29–31^.

### Colle Marino site location and lithology

**Figure 3:**
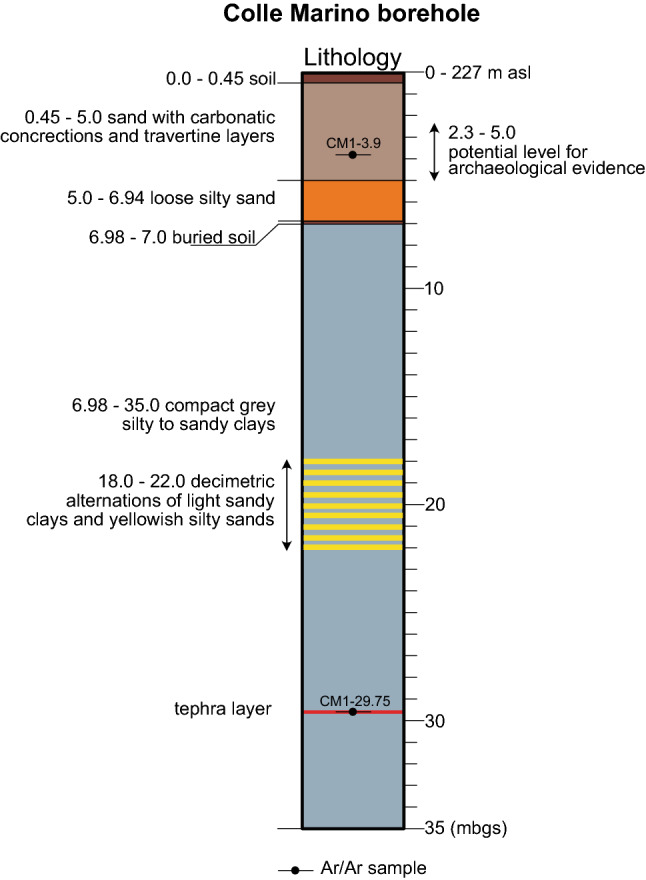
Simplified stratigraphic log of the Colle Marino borehole (CM1). For detailed lithological description and sampling, see Figure S2 in Supplementary Material #1.

Drilling at CM was carried out in the same area (Fig. 1) where the Oldowan-like lithic assemblage^6^ tentatively dated to an age > 0.7 Ma was found. An 8 cm diameter core (from here on, CM1 core) was taken (41°45′32.30″N, 13°07′13.80″E, 227 m elevation; exact location of the drilling site is reported in Fig. S1 of Supplementary Material #1) by standard rotary drilling techniques to a depth of 35 m and the recovered sections lack an azimuthal orientation. The recovered log (Fig. 3; see Fig. S2 for detailed stratigraphy and sampling) includes: the topsoil, from 0 to 0.45 m below the ground surface (bgs); sand with carbonate concretions and travertine layers from 0.45 to 5.0 m bgs, which corresponds to the archaeological horizon^6^; loose silty sand from 5 to 6.98 m bgs, a buried soil from 6.98 to 7 m bgs and compact grey silty- to sandy-clays from 7 to 35 m bgs. Photographs of the cored sediment in the interval 0—9 m bgs are shown in Fig. S3.

In the area surrounding the drilling site^6^, reported the occurrence of a volcanic succession consisting of a lithified tuff at the base, overlain by a several meter-thick pozzolane rich pyroclastic-flow deposit, directly above the sand and travertine horizon. Field work performed for the present study allowed us to recognize the entire volcanic succession as part of the Pozzolane Rosse pyroclastic-flow deposit (456 ± 4 ka^32,33^).

### Field investigations

The investigated site of CM is about 1.5 km from FR and 2 km from CSG (Fig. 1). Chronostratigraphy of the FR site is here reconstructed on the basis of the direct age determination by^12^ and field investigations in the area surrounding the archaeological site. This allowed us to revise the stratigraphic schemes reported by^6^ and^10^, and to frame the stratigraphic setting at FR within the geochronological picture of the Alban Hills volcanic succession^32^. In addition, unpublished detailed stratigraphic logs by A.G. Segre of the two boreholes carried out by^14^ are also used to investigate the subsoil geology.

In order to re-interpret the stratigraphic setting above the palaeontological layer of CSG, we use here data from a large exposed section that we have surveyed at La Vignaccia quarry site, located ca. 0.7 km to the SW (Fig. 1). Here, a variably preserved succession of distal Alban Hills volcanic district pyroclastic-flow deposits unconformably overlies the upper portion of the ca. 10 m thick succession of calcareous silt deposits and travertine layers occurring above the CSG palaeontological horizon. The volcaniclastic succession comprises the primary to sub-primary deposits of the main eruption cycles of the Tuscolano-Artemisio phase, including the Tufo Pisolitico di Trigoria (561 ± 2 ka), Pozzolane Rosse (456 ± 4 ka), Pozzolane Nere (407 ± 3 ka), and Villa Senni (365 ± 4 ka) caldera-forming eruptions^32^.

### Sampling

The clay succession occurring between 5 and 35 m bgs recovered from the CM1 core was sampled for palaeomagnetism using standard 8 cm^3^ plastic cubes, with the arrow on the sampling cube’s bottom pointing up-core, at the IIPU (Anagni, Italy) where the core is stored. We collected 81 samples from the center of the split-core sections, as this is less affected by coring disturbance, with an average spacing of 34 cm. The samples were oriented only with respect to the vertical; the geocentric axial dipole (GAD) field at the latitude of the coring site has an inclination of ± 61°, which makes it feasible to reconstruct palaeomagnetic polarity using only Characteristic Remanent Magnetization (ChRM) inclinations. To minimize sample dehydration, samples were packed in sealed bags and stored in a refrigerated room until they were processed at the INGV in Rome.

In order to provide geochronologic constraints to the sampled interval, the core was initially scrutinized in order to detect macroscopic tephra layers. One sub-primary fallout deposit was observed between 29.70 and 29.80 m bgs and sampled for ^40^Ar/^39^Ar dating. Four additional bulk sedimentary samples were collected at 2.60, 3.90, 5.10 and 11.50 m bgs for detrital sanidine dating (see methods section). Finally, in the fine-grained section between 7 and 35 m bgs, ten samples for biostratigraphic and sedimentological analysis were extracted.

## Laboratory procedures and data analysis

### Palaeomagnetism

Natural and artificial magnetizations were measured using a narrow-access pass-through cryogenic.

magnetometer (2-G Enterprises model 755R) with internal diameter of 4.2 cm, equipped with three DC SQUID sensors (noise level 3 × 10^−9^ A m^2^ kg^−1^), housed in a Lodestar Magnetics shielded room (ambient field < 200 nT).

After measurement of the Natural Remanent Magnetization (NRM), samples were AF-demagnetized at successive peak fields of 5, 10, 15, 20, 25, 30, 40, 50, 60, 80, and 100 mT. The stability of the NRM was assessed using vector component diagrams^34^. ChRM directions were determined using principal component analysis with linear best fits calculated from 3 or more demagnetization steps using the PuffinPlot palaeomagnetic analysis software^35^.

Following AF demagnetization of the NRM, mineral magnetic analyses were conducted on the same set of discrete samples. Low-field magnetic susceptibility (κ) was measured using an AGICO KLY-5 Kappabridge magnetic susceptibility meter with a sensitivity of 2 × 10^–8^ SI.

An anhysteretic remanent magnetization (ARM), which is particularly effective in activating finer magnetic grains, was imparted by using a 0.05 mT direct current (DC) bias field superimposed on a 100 mT peak AF and by translating samples through the AF and DC coil system at 10 cm/s, which is the lowest speed allowed by the control software.

Temperature dependence of the magnetic susceptibility was measured on a few milligrams of powdered samples with a furnace-equipped KLY-5 Kappabridge, in order to determine the characteristic Curie or Néel temperatures of magnetic minerals and discriminate ferromagnetic mineralogy^36^. Samples were measured in air up to a maximum temperature of 700 °C and the thermomagnetic curves were analyzed using the Cureval8 program.

### ^40^Ar/^39^Ar dating methods

Different crystal phases were extracted from five samples of sediment (CSG-13 2A, CM1-2.6, CM1-3.9, CM1-5.1, CM1-11.5), aimed to provide terminus *post-quem* (maximum age) to the time of deposition, and from one sample of a primary fallout deposit (CM1-29.75).

Samples CM1-2.6, CM1-3.9, CM1-5.1, CM1-11.5 were prepared (crushing, sieving, cleaning, and handpicking) at the Laboratoire des Sciences du Climat et de l’Environnement of Gif-sur-Yvette (France) facility using the same procedure as in^11^. Unfortunately, samples CM1-2.6, CM1-5.1, CM-11.5 did not yield K-bearing mineral phases suitable for ^40^Ar/^39^Ar dating. Pristine sanidines and few leucites ranging in size between 1 mm and 300 µm were otherwise found in the sample CM1-3.9.

The selected minerals were irradiated for 120 min in the Cd-lined, in core CLICIT facility of the Oregon State University TRIGA reactor (IRR CO007). Interference corrections were based on the nucleogenic production ratios given in^37^. After irradiation, individual crystals were transferred into a copper 134 pits sample holder placed into a differential vacuum Teledyne Cetac window connected to the extraction line. Minerals were fused one by one using a 100 Watts Teledyne Cetac CO_2_ laser during 15 s at 2.3 W. Before fusion, each crystal underwent a 10 s long sweeping at 0.2 W to remove unwanted gas trapped superficially. Extracted gas was purified by a SAES GP50 cold getter for 90 s and then for 210 s by two hot SAES GP50 getters. The five Argon isotopes (i.e., ^40^Ar, ^39^Ar, ^38^Ar, ^37^Ar and ^36^Ar) were measured using a NGX 600 mass spectrometer equipped with 9 ATONA Faraday cups and an electron multiplier. Technical specifications and performances of the NGX 600 ATONA detector array are presented in detail by^38^.

^40^Ar, ^39^Ar, ^38^Ar, ^37^Ar and ^36^Ar isotopes were collected simultaneously while the ^37^Ar was measured in a second time. In the first run, ^40^Ar, ^39^Ar and ^38^Ar were measured simultaneously on 3 ATONA and ^36^Ar using the electron multiplier. Then, just following this first run ^37^Ar was measured using the electron multiplier after peak switching. Each isotope measurement corresponds to 15 cycles of 20-s integration time. Peak intensity data were reduced using ArArCALC V2.4^39^.

Neutron fluence J for each sample was calculated using co-irradiated Alder Creek sanidine standard ACs-2 at 1.1848 Ma^40^ according to the K total decay constant of^41^ (λ_e.c._ = (0.580 ± 0.007) × 10^−10^ yr^−1^ and λ_β¯_ = (4.884 ± 0.049) × 10^−10^ yr^−1^). To determine the neutron flux of IRR CO007 flux monitors were placed in small pits framing the sample. Two standards from 3 pits around the unknown were measured to calculate CM1-3.9 J-value (J = 0.0005519 ± 0.00000055).

Mass discrimination was monitored by analysis of 30 air pipettes of various beam sizes to verify the detectors linearity. These measurements are done automatically during the nights before and after the unknown measurements. Discrimination is calculated according to the ^40^Ar/^36^Ar ratio of 298.56^42^. Procedural blank measurements were achieved after every four unknowns. For typical 5 min time blank backgrounds are between 1.7 10^−4^ V and 2.0 10^−4^ V for ^40^Ar and 65 cps for 36 Ar (9.5 10^−7^ V equivalent).

Separates from sample CSG-13 2A and CM1-29.75 were co-irradiated with the 1.1864 Ma Alder Creek sanidine standard^43^ at the Oregon State University TRIGA reactor in the Cadmium-Lined In-Core Irradiation Tube. Single crystal fusion analyses were performed at the WiscAr laboratory at the University of Wisconsin-Madison using a 60 W CO_2_ laser and a Noblesse multi-collector mass spectrometer following^43^.

Results for all the dated samples are reported with 2σ analytical uncertainties according to the respective laboratory standards (see above). Difference due to the two different calibrations affects the third decimal figure and are negligible within the aims of this work. Full analytical data are reported in Supplementary Material #2.

### Sedimentological and biostratigraphic analysis

Samples were oven-dried at 50 °C, weighed, and subsequently washed and sieved through a 63 μm mesh and dried again at 50 °C. Separates were observed under an optical microscope LEICA M165 and selected fossils were analyzed with the Scanning Electron Microscope HITACHI TM3030 Plus. The semiquantitative analyses carried out on the microfossil content, including ostracods, and small bivalves, led to the rough indication of the occurrence of the most indicative taxa, in order to identify palaeoenvironmental changes.

### Petrographical and SEM analysis

Mineral compositions were analysed at the CNR-Istituto di Geologia Ambientale e Geoingegneria (Rome), with a Cameca SX50 electron microprobe equipped with five wavelength dispersive spectrometers (WDS). Quantitative compositional analyses were performed using 15 kV accelerating voltage and15 nA beam current. As standards we employed metals for Mn and Cr, jadeite for Na, wollastonite for Si and Ca, orthoclase for K, corundum for Al, magnetite for Fe, rutile for Ti and periclase for Mg. Counting times were 20 s for elements and 10 s for backgrounds. Light elements were counted first to prevent loss by volatilization. The PAP correction method was used.

## Results

### Magnetic properties

**Figure 4:**
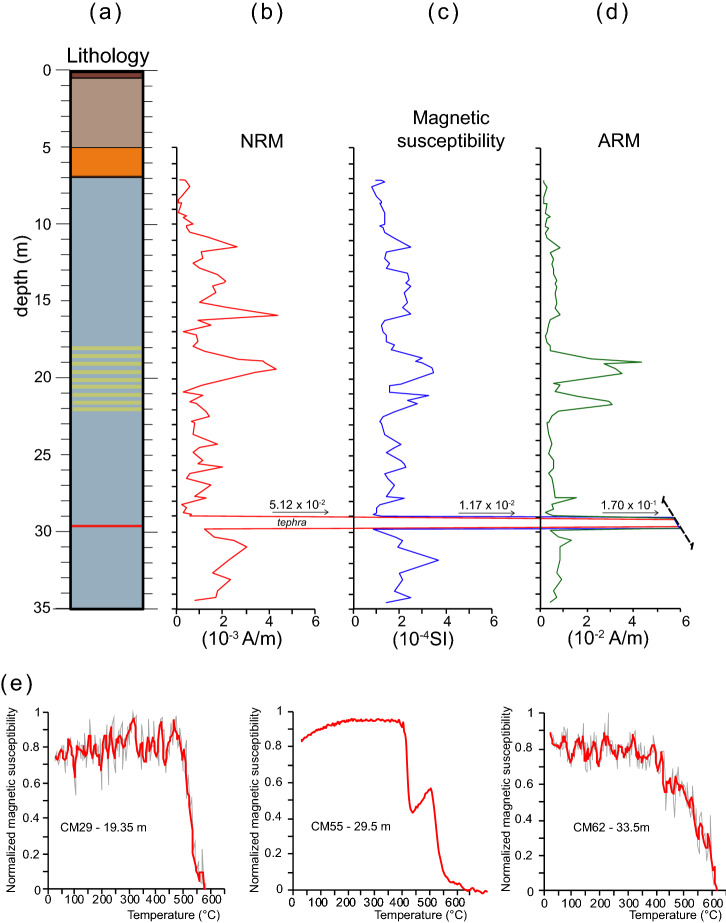
Mineral magnetic data for CM1. (**a**) lithology, (**b**) stratigraphic variations of NRM intensity, (**c**) κ and (**d**) ARM. (**e**) Temperature dependence of κ (up to 700 °C) for three representative samples: CM29 (19.35 m), CM55 (29.5 m), CM62 (33.5 m). For samples CM29 and CM62 a three-point moving average is applied (red curves).

Within CM1 core, NRM, κ and ARM do not show significant changes with a single peak in correspondence of a thin tephra layer at 29.75 m bgs (Fig. 4). NRM intensity values range between 3.89 × 10^–5^ A/m and 4.36 × 10^–3^ A/m with an average of 1.22 × 10^–3^ A/m. The single sample at the tephra level has an intensity of 5.12 × 10^–2^ A/m. Down-core variations of κ are associated with similar changes in ARM intensity which suggests that these fluctuations are mainly controlled by changes in magnetic mineral concentration. κ values range between 8.22 × 10–5 SI and 3.69 × 10^–4^ SI with an average of 1.78 × 10^–4^ SI (data are reported in Supplementary Material #3).

The increase in k and ARM in the interval between 18 and 22 m corresponds to a portion of the core characterized by decimetric alternations of light sandy clays and yellowish silty sands.

At the tephra level κ = 1.17 × 10^–2^ SI. ARM values range between 1.31 × 10^–3^ A/m and 4.32 × 10^–2^ A/m with an average of 7.44 × 10^–3^ A/m. The single sample at the tephra level has an intensity of 1.70 × 10^–1^ A/m.

Thermomagnetic curves on three selected samples (Fig. 4e) were produced via progressive heating. Samples CM29 (19.35 m bgs) and CM62 (33.50 m bgs) show a noisy susceptibility versus temperatures curves due to a weak κ but it has been still possible to observe a major drop occurring at ca. 580 °C (temperatures corresponding to the inflection point during the decrease in susceptibility) which indicates the ubiquitous presence of Fe spinels with a composition similar to magnetite. A more complex mineralogy occurs for tephra sample CM55 (29.5 m bgs) where a decrease at ca. 415 °C is followed by a second drop at ca. 530 °C which indicate the presence of unstable cation deficient titanomagnetite exsolved into Ti–rich phases upon heating in air^44^.

### Demagnetization behavior and polarity zonation

**Figure 5:**
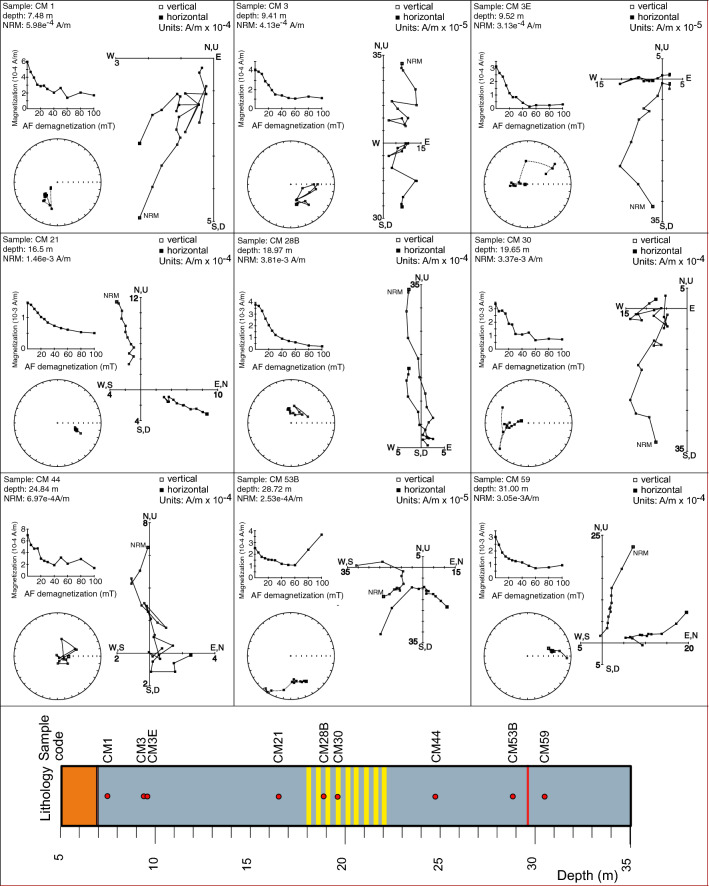
AF (alternating field) demagnetization behavior for 9 representative samples from CM1. For the vector component diagrams, open (closed) symbols represent projections onto the vertical (horizontal) plane. The stereoplots are equal-area projections, with solid (open) symbols representing points projected onto the lower (upper) hemisphere. The cores were not azimuthally oriented; declinations are reported in the laboratory coordinate system with respect to the split face of the core. Plots were produced using PuffinPlot^35^.

Stepwise AF demagnetization enabled isolation of the ChRM component for 63 (78%) of the samples analyzed. Most of the samples are characterized by noisy demagnetization paths in orthogonal vector diagrams but are sufficiently good to define a magnetic polarity zonation. The unstable behavior is reflected in a with maximum angular deviation (MAD) value comprised between 1.0° and 20.6°, with an average of 7.9°. Some samples, mainly from the sandy horizons, are characterized by aberrant behavior (Fig. 5) due to the fact that the deposition of large particles would be controlled by gravitational rather than magnetic forces. Thus, their orientation could not be expected to represent the geomagnetic field at or near the time of deposition and have been discarded.

In order to avoid the shallowing bias which can be introduced when taking the arithmetic mean of the inclination values, mean ChRM inclinations were computed separately for normal and reverse polarity populations using a maximum likelihood method^45^ as implemented in the PuffinPlot application^35^. The normal polarity samples and the reverse polarity samples have a mean inclination of 57.8° (N = 13; a95 = 21.8°) and -55.6° (N = 49; a95 = 5.6°), respectively. These values are not significantly different from antipodal at the 95% confidence level, affording a positive reversal test, and suggesting that the mean directions could indeed represent a reliable polarity record.

**Figure 6:**
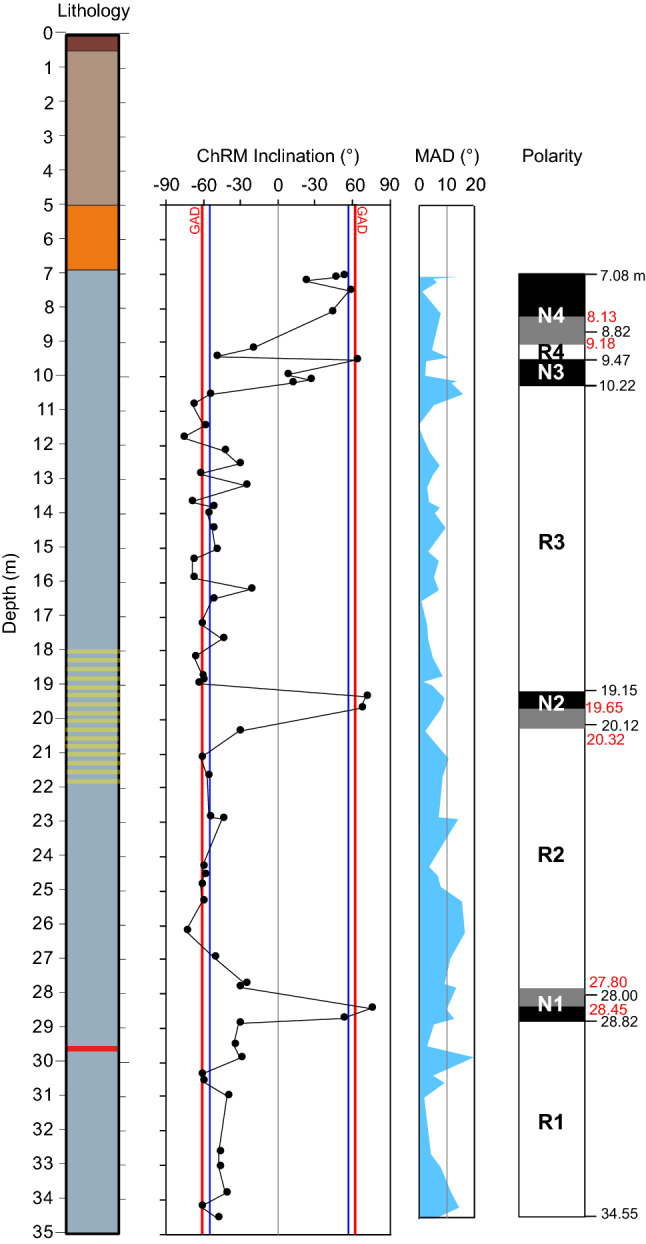
Lithology (symbols as in Fig. 3), downcore variations of ChRM inclination, MAD, and magnetic polarity zonation (black = normal polarity and white = reverse polarity).

The ChRM inclinations enable delineation of eight magnetozones, which are defined using at least two consecutive samples with inclinations distinctly different from neighboring intervals (Fig. 6). Boundaries between magnetozones are placed at the mid-point between successive samples of opposite polarity. Starting from the bottom, the lower 5.73 m (from 34.55 to 28.82 m bgs) of the magnetic polarity record has reverse polarity (magnetozone R1); the lower boundary of this magnetozone was not sampled. Magnetozone N1, from 28.82 to 28.00 m bgs, has two samples with stable normal polarity. The boundary between magnetozones N1 and R2 lies within an 0.65 m interval (indicated in grey in Fig. 6). Above magnetozone N1, a reverse polarity interval (R2) extends from 28.00 to 20.12 m bgs and it is followed, from 20.12 to 19.15 m bgs, by magnetozone N2 defined by two samples with stable normal polarity. The boundary between magnetozones R2 and N2 lies within an 0.67 m interval with no samples. The reversed polarity magnetozone R3 is well defined and extends from 19.15 and 10.22 m bgs. The uppermost 10.22 m of the studied sequence is dominated by two normal polarity intervals (N3 and N4) separated by a reverse polarity interval (R4), from 9.47 to 8.82 m bgs. Samples at 9.52 (normal polarity) and 9.41 m bgs (reversed polarity) define the boundary of magnetozone N3 with R4. The boundary position between R4 and N4 has a range of uncertainty of about 1 m due to the lack of samples in this interval with acceptable demagnetization behaviors useful for defining the ChRMs. Data are reported in Supplementary Material #3.

### ^40^Ar/^39^Ar dating

#### CM1-3.9

**Figure 7:**
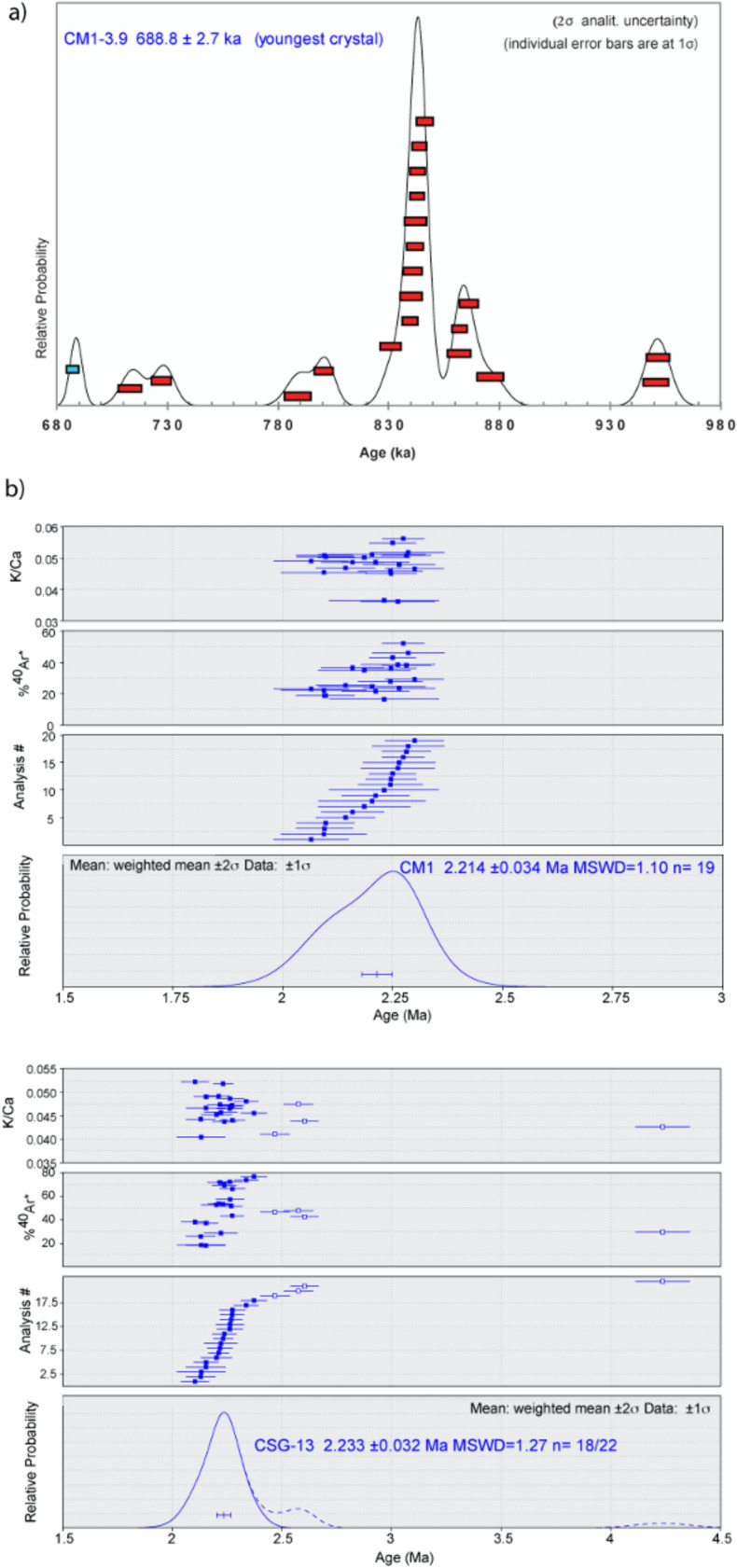
Relative probability diagrams showing the results of the 40Ar/39Ar dating experiments performed at LSCE (**a**) and at the Wiscar Laboratory (**b**). Full analytical data are reported in Supplementary Material #2, #3.

Twenty-one sanidine crystals were dated and provided very scattered ages ranging between about 955 and 689 ka (see probability diagram in Fig. 7a and Supplementary Material #2). The youngest crystal is dated to 688.5 ± 2.7 ka (2σ analytical uncertainty). A population constituted by 9 on 21 dated crystals is dated to 843.3 ± 1.6 ka (MSWD = 1.20; P = 0.30, 2σ analytical uncertainty including J value).

#### CM1-29.75

Nineteen of 22 K-bearing plagioclase crystals give a weighted mean ^40^Ar/^39^Ar age of 2.214 ± 0.034 Ma (Fig. 7b). Three dates are markedly younger and are excluded from the weighted mean calculations. These younger dates may be due to alteration or Ar loss.

#### CSG-13 2A

Eighteen of 22 single plagioclase fusions from sample CSG-13 2A give a weighted mean ^40^Ar/^39^Ar age of 2.233 ± 0.032 Ma (Fig. 7b). Four older dates are excluded from the weighted mean calculations and are interpreted to be antecrysts or partially degassed xenocrysts.

### Age model

**Figure 8:**
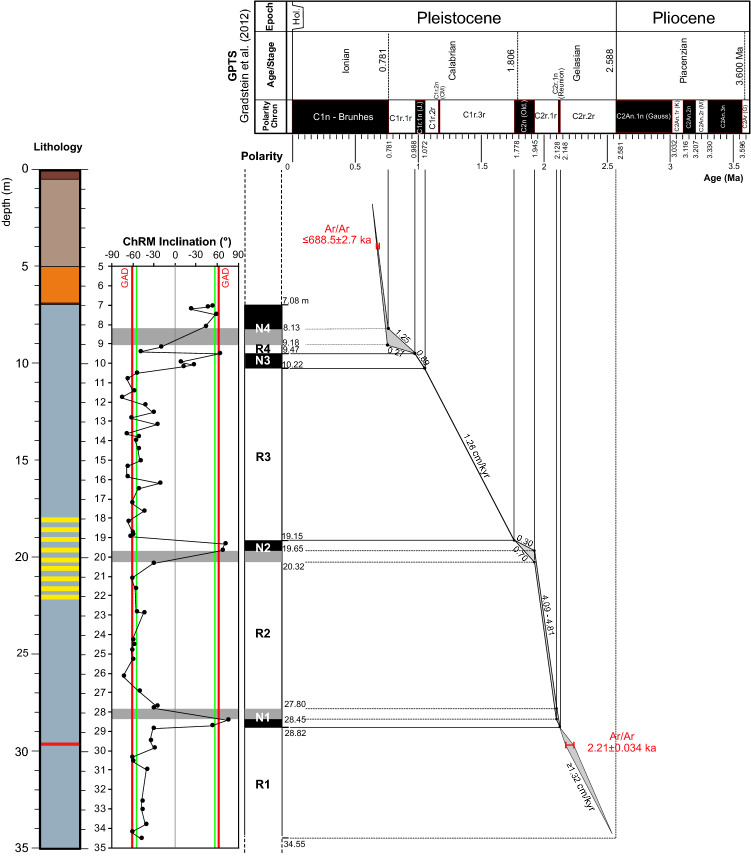
Correlation of the new magnetic polarity zonation from CM1 with the GPTS of^46^.

We refer the geomagnetic polarity time scale (GPTS) of^46^ to develop an age model for the studied interval. Our magnetostratigraphic interpretation is presented in Fig. 8 along with ^40^Ar/^39^Ar ages from both primary and reworked volcanic materials and the uncertainties associated with each datum. An ^40^Ar/^39^Ar age of 2.21 ± 0.034 ka (2σ analytical uncertainty) for a tephra layer at 29.75 m bgs constrains correlation of magnetozone R1 to Chron C2r.2r (2.148–2.588 Ma). The youngest sanidine crystal extracted from a sediment sample at 3.9 m bgs, is dated to 688.5 ± 2.7 ka providing a maximum age for this horizon and constrains correlation of magnetozone N4 to Chron C1n (Brunhes; 0–0.781 Ma). This is in agreement with the absence of primary volcanic deposits ascribable to the Alban Hills and Monti Sabatini volcanic districts, confirming that the top of the sequence, from 0 to 3.9 m bgs, is older than ~ 0.6 Ma.

The two ^40^Ar/^39^Ar ages and the absence of products of the paroxysmal volcanic activity, support a one-by-one correlation of observed magnetozones with each polarity of the GPTS from Chron C1n to C2r.2r with variable sedimentations rate up to 4.81 cm/kyr.

### Biostratigraphy

The investigated clayey succession is remarkably barren, with a total absence of any biogenic fraction in all the samples collected between 12 and 35 m bgs, as well as in one sample collected at the very top, 7.5 m bgs (Table 1).

**Table 1:** Composition of the mineralogical and biogenic fraction > 63 μm in the investigared samples.

Sample-depth	Terrigenous fraction	Biogenic fraction
CM 1 – 7.2	quartz+++, feldspar + , biotite-, oxidised carbonates concrections	Barren
CM 1 – 9.5	quartz+++ feldspar + , pyroxene-, biotite-,	Ostracods (Candona sp. and Cypridea torosa), rare undetermined Bivalves, calcified remains of charophyte cortex (Characee)
CM 1 – 12.7	quartz + + + , feldspar + , pyroxene-, biotite-, white pumice + +	Barren
CM 1 – 15.50	quartz + + + , feldspar + + , biotite +	Barren
CM 1 – 18.9	quartz + + + , feldspar + , pyroxene + , biotite + , yellowish pumice + +	Barren
CM 1 – 21.6	quartz + + + , feldspar + , biotite + + , pyroxene + , yellowish pumice + +	Barren
CM 1 – 26.60	quartz + + + , feldspar + + , biotite +	rare vegetal fragments
CM 1 – 29.3	quartz + + + , feldspar + , amphibole + + , pyroxene + , biotite + , pyrite +	Barren
CM 1 – 33.00	quartz + + + , feldspar + , biotite + + , pyroxene + +	Barren
CM 1 – 35	quartz + + + , feldspar + , pyroxene-, biotite-, pyrite-	barren

A scanty ostracod assemblage has been found only in a sample at 9.5 m bgs, represented by *Ilyocypris gibba* and *Candona* sp., along with a few undetermined bivalve shells (Fig. S4a in Supplementary Material #1). The occurrence of *Ilyocypris gibba* with *Candona* sp. indicates fresh—oligohaline inland water body, with a mud substrate^4,47^. However, idiosyncratic calcified Charophytes thalli have been also observed in this sample (Fig. S4b).

Indeed, Charophytes (Characeae), are macroscopic green algae occurring mainly in the littoral zone of lakes. Photosynthetic activity of charophytes leads to precipitation of autochthonous carbonates, which significantly contribute to lacustrine sedimentation. The occurrence of most charophytes is limited between about 1 m to 10 m of depth in fresh water bodies with clear, alkaline waters, while they become rare along with increasing trophy level and decreasing light availability (Garcia, 1994 in^48^).

### Tephrostratigraphic and biochronologic data

The tephra at 29.75 m bgs (sample CM1-29.75) contains abundant amphibole and plagioclase crystals. Amphibole consists of submillimeter, euhedral to subeuhedral, deep green crystals including titanomagnetite. According to the classification scheme of^49^, the composition of amphibole is edenite with an Mg# restricted to a nearly constant value of 60–64. Plagioclase is found as submillimeter, euhedral to subeuhedral crystals, including apatite and is characterized by continuous normal zoning with anorthite (An_47–57_) content variable from core-to-rim.

The mineral chemistry of the tephra is typical of calcalkaline rocks. In particular, the Mg# of amphibole and the anorthite content of plagioclase indicate a crystallization from an andesite magma^50^. The calcalkaline rocks underlying the Phlegrean Fields volcanic suites show similar K/Ar age^51^ of the CM1-29.75 tephra. Nevertheless, buried Campanian volcanics show porphyritic textures with phenocrysts of zoned plagioclase but without amphiboles. The latter phase occurs in the calcalkaline rocks of Capraia island but are much older (> 4.6 Ma;^52^).

**Figure 9:**
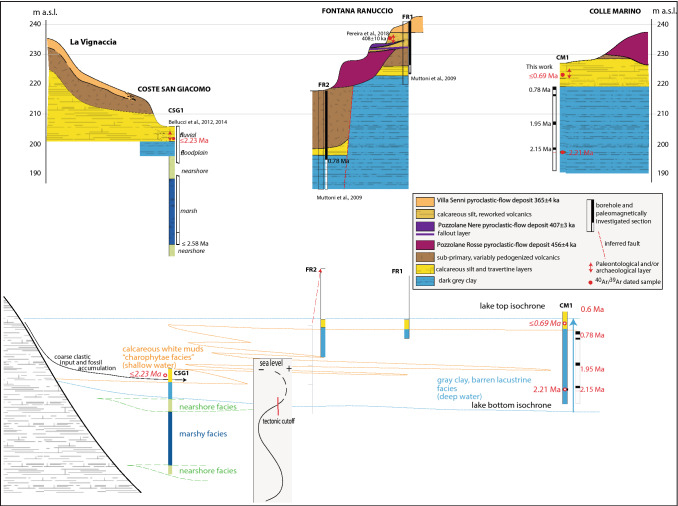
(**a**) Reconstruction of the stratigraphic setting of the investigated area through the cross-correlation of the borehole data achieved in the present study and in previous work at Colle Marino, Fontana Ranuccio and Coste San Giacomo. (**b**) Sketch showing the environmental evolution of the Latin Valley in the interval 2.6–0.6 Ma, after restoration of the original elevation of borehole FR2 before tectonic dislocation (see text for explanations). Drawings by FM.

The CSG-13 2A sedimentary sample contained a significant amount of plagioclase crystals; 18 out of 22 yielded a homogeneous population age of 2.233 ± 0.032 Ma, virtually undistinguishable from that of 2.214 ± 0.034 Ma yielded by the primary tephra sample CM1-29.75, suggesting the sub-primary origin for the volcanic material included in the sediment. Indeed, photographs of the original excavation from which sample CSG-13 2A was collected (Fig. S5 in Supplementary Material #1) show the occurrence of cross-laminated, dark grey sands, which may be regarded as deriving from the reworking of a primary fallout deposit emplaced shortly before, as suggested by the abundant, homogeneous plagioclase crystals population recovered in the sedimentary sample. Consistent with this inference, the palaeontological layer of CSG occurs at similar, yet slightly higher elevation with respect to the tephra layer recovered from CM1 (Fig. 9). Therefore, the age of 2.233 ± 0.032 Ma can be considered the same of the faunal assemblage of Middle Villafranchian age recovered at CSG^4^.

The CSG faunal unit belong to the MNQ17b biozone the reference site of which is located in the French *Massif Central* (Senèze) and is dated by ^40^Ar/^39^Ar between 2.10 and 2.18 Ma^53^. The age obtained for the fossil-bearing horizon at CSG suggests an earlier arrival of some species in Italy than in France and give a minimum age of 2.23 Ma for the base of the MNQ 17b biozone.

## Discussion

The chronostratigraphic constraints achieved for the sedimentary succession recovered from the borehole and documented in the field at the three palaeontological/archaeological sites show that these successions cover three distinct, partially overlapping, time intervals spanning ca. 2.6 through 0.36 Ma (Fig. 9). The tephra recovered at CM1 provides exact correlation with the fossil-bearing horizon of CSG, where a nearly homogeneous K-plagioclase crystal population extracted from a sand sample yielded an identical age, within the analytical errors, of 2.233 ± 0.032 Ma.

Remarkably, the clay section underlying the fossiliferous horizon at CSG1 borehole, between 198 and 193 m a.s.l., shows lithological and biostratigraphic features very similar to the clay interval underneath the tephra layer from the CM1 borehole. The clay section was described by^4^ as part of a floodplain facies association, characterized by dominant greenish/bluish, fine-grained, laminated sediments, with common CaCO_3_ nodules, rootlets, and very scanty organic material. Few ostracods (*Pseudocandona rostrata* with subordinate *Candonopsis* juv.) have been recovered only in the upper, coarsening upward part of the facies association (201–198 m a.s.l.), which makes transition to the fluvial-channel facies association (206—201 m a.s.l.) in which a Middle Villafranchian vertebrate faunal assemblage occurs^4–10^.

In contrast, the lower portion of the clay section recovered from 193 to 163 m a.s.l. at CSG1 displays distinct features from those detected from 220 to 192 m a.s.l. (from 7 to 35 m bgs) at CM1, consistent with its different, older age range.

By combining the abundant palynological and ostracod records with facies analysis of the sedimentary succession^4^, recognized two near-shore facies associations at the top and at the bottom of the cored sediments, separated by a thick marsh facies association, with abundant organic-rich (lignite) layers.

No lower geochronologic boundary is achievable for the CSG1 clay section. A minimum age ≤ 2.58 Ma can be inferred based on the reversed polarity yielded by the whole analyzed set of samples after^5^, with the exception of two. Still, once the high sedimentation rate deriving from this assumption is taken into account, the possibility that the lowest normal polarity sample occurring at the base of the clay section may represent the top of the Gauss normal Chron C2An.1n (2.581 Ma) cannot be discarded.

The low elevation at which the Brunhes-Matuyama reversal is found in borehole FR2, combined with the large offset affecting the lithological boundary between the white calcareous silt and the grey lacustrine clay from cores FR1 and FR2^14^, suggests the occurrence of fault displacement (Fig. 9a). In addition, both the large thickness of the Pozzolane Rosse pyroclastic-flow deposit (456 ± 4 ka;^32^) and the occurrence of a deeply zeolitized basal portion point to deposition within an incised palaeovalley corresponding to a tectonic structure (“graben”) which would host the present Fosso delle Mole streambed (Fig. 1). Similarly, a thick succession of reworked volcanic material, older than the Pozzolane Rosse emplaced in fluvial-lacustrine setting at FR2, constrains the tectonic activity within the 600–450 ka time interval.

After the abovementioned tectonic offset is restored, the stratigraphic setting around 600 ka is reconstructed in Fig. 9a, while a sketch of the palaeogeographic conditions controlling the geologic evolution of this area since 2.6 Ma is shown in Fig. 9b.

**Figure 10:**
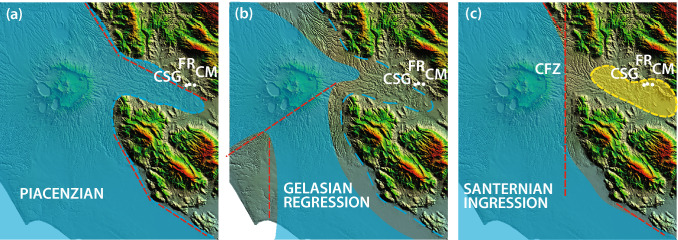
Paleogeographic sketches showing the sea ingression and regression phases occurring in the investigated area 2.5 Ma through 1.8 Ma. See text for explanations. DEM images used with permission of the Istituto Nazionale di Geofisica e Vulcanologia, Rome.

Biostratigraphic features of the lower clay section recovered at CSG1 reflect the regional relative sea-level oscillations of the Tyrrhenian Sea basins during the Piacenzian (Fig. 10;^54^ and references therein). The nearshore deposits found at the base and at the top of this succession testify approaching of the coastline during the Piacenzian ingression 3.6 through 2.6 Ma, and during the early stages of the Gelasian/Santernian ingression, since 2.2 Ma. This evolution reflects the palaeogeographic conditions of the Latin Valley, which was an eastward stretching, deep gulf of the Tyrrhenian Sea during the two ingressive phases, while it became an estuarine, large alluvial plain during the Gelasian regressive phase, when the thick marshy facies association was deposited.

However, as suggested in^54^, the Santernian ingression in the Latin Valley was aborted shortly after 2.2 Ma, in consequence of a tectonic "cutoff" of this engulfed region. Very likely, this process was due to the re-activation of a N-S strike-slip fault (Fig. 10c; Cisterna Fault Zone—CFZ^55^), which created a structural threshold hindering the sea ingression, and forming a large lacustrine basin occupying the whole Sacco Valley (Figs. 1, 2, 10).

Previous literature^56,57^ interpreted the eastward "inversion" of the drainage in the Sacco Valley, from an original westward direction towards the Aniene and Tiber rivers catchment basins, has due to the emplacement of large thickness of Alban Hills pyroclastic-flow deposits since 450 ka. Palaeogeographic reconstruction based on the CSG1 and CM1 borehole data discussed here suggest that a significant change in the hydrographic catchment occurred much earlier, around 2.2 Ma, creating the conditions for the successive inversion of the river flow direction.

Indeed, rather than evolving towards a marine environment, like what happened along the northern stretch of the "Santernian coastline" (^54,58^ for a detailed discussion) (Fig. 10), a continental, lacustrine environment characterized the Sacco Valley from 2.2 through 0.6 Ma, at the least.

The lacustrine deposits spanning this age interval represent the entire, 35 m thick sedimentary succession recovered between 192 and 227 m a.s.l. at the CM1 borehole, and they constitute the uppermost 12 m, from 195 to 206 m a.s.l., at CSG1. However, the geologic section exposed at La Vignaccia allows a correlation of the thick succession of calcareous silt with frequent carbonate encrustations cropping out between 210 and 225 m a.s.l. with the coeval lacustrine succession in CM1. The top of the calcareous silt deposit reaches an elevation of 225 m a.s.l. in La Vignaccia, thus leading to estimate a thickness of ca. 20 m for this pack of homogeneous lacustrine sediments overlying the fossiliferous horizon of CSG. The “volcanic” age boundary at ~ 0.6 Ma shows that this sedimentary sequence accumulated during a time span of ca. 1.5 Myr, since 2.21 Ma. The thick suite of primary to deeply reworked volcanic materials exposed at La Vignaccia is correlated to the deposit described in the stratigraphic log of FR’s boreholes 1 and 2, occurring between 232–227 and 217–202 m a.s.l., respectively. This sequence of reworked volcanics rests on the eroded sedimentary substrate, while its base deepens to the east, towards the depocenter of the lacustrine basin, that is, where FR and CM are located. In general, this correlation allows us to understand the palaeoenvironmental features of the lacustrine basin. With a deep depocenter near CM1, barren clay sediments accumulated in this area, and a peripheral, shallow coastal setting, characterized by clear waters allowing photosynthetic activity of charophytes, led to precipitation of autochthonous carbonates that substantially contributed to the lacustrine sedimentation.

These environmental conditions are framed within a significant climate event which possibly is the driving factor of the Gelasian/Santernian ingression and to the dispersal events that led into Europe African taxa such as, among others, *Homo* sp.^59^. Moreover, the CSG faunal assemblage^4^ testifies the early-middle Villafranchian mammal turnover, characterized by the dispersal in Europe of species that adapted to open environments such as elephants and horses^60^.

Since ~ 0.8 Ma, progressive shallowing of the lake resulted in an eastward "progradation" of the "Charophyte facies". Consistent with appearance of Charophytes and the abundant carbonate precipitation testified by the diffuse nodules occurring in the uppermost 3 m of the clay succession, a sharp transition to white, calcareous sandy silts occurs at 220 m a.s.l. at CM1 (Fig. 9). The transition is marked by a decimeter-thick interval of alternating thin layers of white carbonatic silt and dark-brown, organic-rich clay sediment. At 200 m a.s.l. the calcareous silt horizon passes upwards (the lithologic passage was not recovered in the core due to the partial loss of sampling caused by the unconsolidated features of the silt deposits) to yellow sands with abundant carbonate encrustations and travertine layers, in which two red soils occurs. The youngest sanidine crystal out of 21 extracted from a sediment sample collected within this horizon (CM1-3.9) provided a maximum age of 688.8 ± 2.7 ka for this layer.

We interpret the progressive shallowing and temporary emersion of the lacustrine basin as the combined effect of the sedimentary filling and regional uplift that occurred since 0.8 Ma and culminated around 0.6 Ma, in coincidence with the onset of the volcanic activity in the Roman Magmatic Region. In this context, the lacustrine basin of the Sacco Valley can be regarded as a fault-controlled, subsiding basin, within the overall uplifting regime of the Apennine chain during Middle Pleistocene times (e.g.,^61–63^).

This location, which contains the earliest evidence of human occupation in central Italy (e.g.,^64,65^) is characterized by large lacustrine basins and surrounding alluvial plains with prairies and grassland and mild climatic conditions, and represent an ideal environment for game and their hunters.

## Conclusions

The present study provides accurate geochronologic constraints to the sedimentary succession recovered from a drilling at CM and unambiguously correlate it with those previously investigated at the nearby fossiliferous sites of CSG and the archeological site of FR. The main findings of this study are:

- Correlation of the sequence of magnetozones with the GPTS is constrained by ^40^Ar/^39^Ar ages obtained from both in situ and reworked volcanic materials.
- The chronostratigraphic constraints show that the investigated successions cover three distinct time intervals, partially overlapping, spanning ca. 2.6–0.36 Ma.
- The lower clay succession recovered at CSG records two regional sea-level oscillations corresponding to the "Piacenzian" (i.e., 3.6–2.6 Ma) and to the early "Gelasian/Santernian" (i.e., 2.2–1.5 Ma) ingressions.
- The second ingressive phase is abruptly aborted around 2.2 Ma, as attested by the lack of marine deposits of Santernian age (1.8–1. 5 Ma) in the CM1 borehole.
- A large lacustrine basin occupied the Latin Valley since 2.2 Ma providing an environment favorable to the dispersal of several "African" taxa which, among others, constitute the Middle Villafranchian Faunal Unit of CSG which is dated in the present work at 2.233 ± 0.032 Ma.
- The age of CSG fossiliferous horizon slightly pre-dates previous assessment at the French type-locality of the MNQ 17b biozone, suggesting an earlier occurrence of the African taxa in Italy than in France.
- Starting from 0.8 Ma, the progressive shallowing and temporary emersion of the large lacustrine basins created favorable conditions for early hominin occupation of the area, as it is possibly documented by the lithic assemblages from CM.
- The CM layer dated at about 0.7 Ma, tentatively suggested as being the origin of the lithic collection found in the site, could provide a strong reference for the pre-Acheulean technocomplexes in Mediterranean Europe.
- The human occupation of this region reaches a climax around 0.4 Ma as constrained by the chronostratigraphic framework reconstructed here for the FR site, consistent with geochronologic data from recent literature achieved at this site and several other sites throughout the Latin Valley, spanning 0.41–0.35 Ma.

These results contribute to better interpretation of the hominin migratory dynamics and the factors that influenced the location and spatial distribution during the early stages of occupation in these regions.

## Supplementary Information

Supplementary Information 1.

Supplementary Information 2.

Supplementary Information 3.

Supplementary Information 4.
