# Supplementary material for: Environmental evolution, faunal and human occupation since 2 Ma in the Anagni basin, central Italy
Source: Sci Rep. 2021 Mar 29;11:7056. doi: 10.1038/s41598-021-85446-5 (PMC8007579; doi:10.1038/s41598-021-85446-5)
Supplement: Supplementary file 1 — Supplementary Information 1. [file 41598_2021_85446_MOESM1_ESM.pdf]

Environmental evolution, faunal and human occupation since 2 Ma in the Anagni basin, central Italy

Fabio Florindo[1,2*], Fabrizio Marra[1], Diego E. Angelucci[3], Italo Biddittu[4], Luciano Bruni[4], Federico Florindo[5], Mario Gaeta[6], Hervé Guillou[7], Brian Jicha[8], Patrizia Macrì[1], Caterina Morigi[9], Sebastien Nomade[7], Fabio Parenti[4,10], Alison Pereira[11,12], Stefano Grimaldi[3,4]

1 Istituto Nazionale di Geofisica e Vulcanologia, Rome, Italy
2 Institute for Climate Change Solutions, Via Sorchio snc, 61040 Frontone, Italy
3 Dept. of Humanities, University of Trento (Trento, Italy)
4 Istituto Italiano di Paleontologia Umana (Anagni, Italy)
5 Sapienza Università di Roma, Piazzale Aldo Moro 5, 00185, Roma, Italy
6 Sapienza Università di Roma, Dipartimento di Scienze della Terra, Piazzale Aldo Moro 5, 00185, Roma, Italy
7 Laboratoire des Sciences du Climat et de l'Environnement. LSCE/IPSL, UMR CEA-CNRS-UVSQ 8212. CEA Saclay, Bat 714. Chemin de Saint Aubin - RD 128 F-91191 Gif sur Yvette France
8 Department of Geoscience, University of Wisconsin-Madison, USA
9 Department of Earth Sciences, University of Pisa, Via S. Maria 53, 56126 Pisa, Italy
10 Universidade Federal do Paraná, Curitiba, Brazil
11Université Paris-Saclay, CNRS Laboratoire GEOPS, Orsay, France
12Département Hommes et environnements, Muséum national d'Histoire naturelle, Paris, France

*corresponding author: fabio.florindo@ingv.it

Supplementaery Material #1
Stratigraphic information

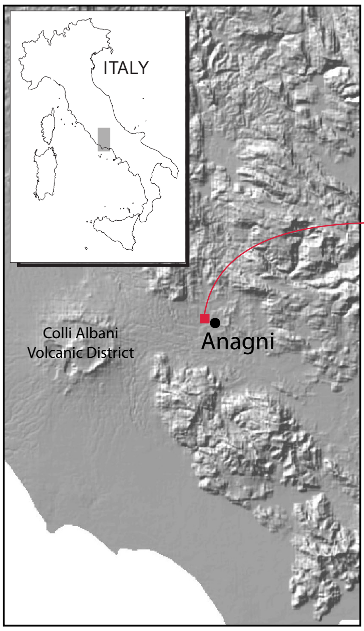

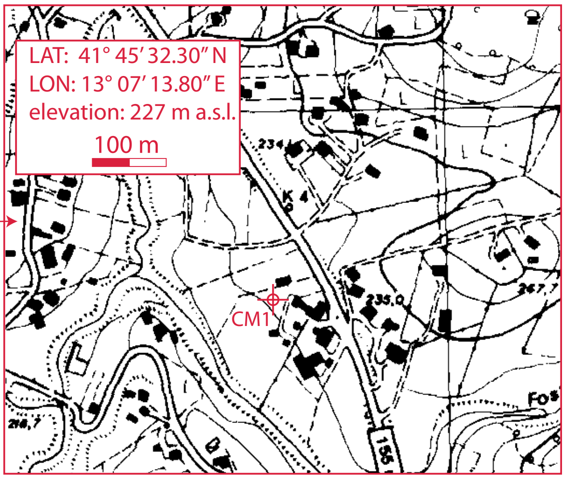

Figure S1 - 1.10.000 topographic map showing CM1 borehole location. (Carta tecnica regionale 10K formato TIFF, 1990-91, Regione Lazio, available under the Creative Commons Attribution license at: http://dati.lazio.it/weblist/cartografia/prodotti/1990_1991_CTR_10K_TIF/)

Digital Elevation Map (TINITALY/01 square WA 6570, property of the Istituto Nazionale di Geofisica e Vulcanologia, Rome, http://tinitaly.pi.ingv.it/)

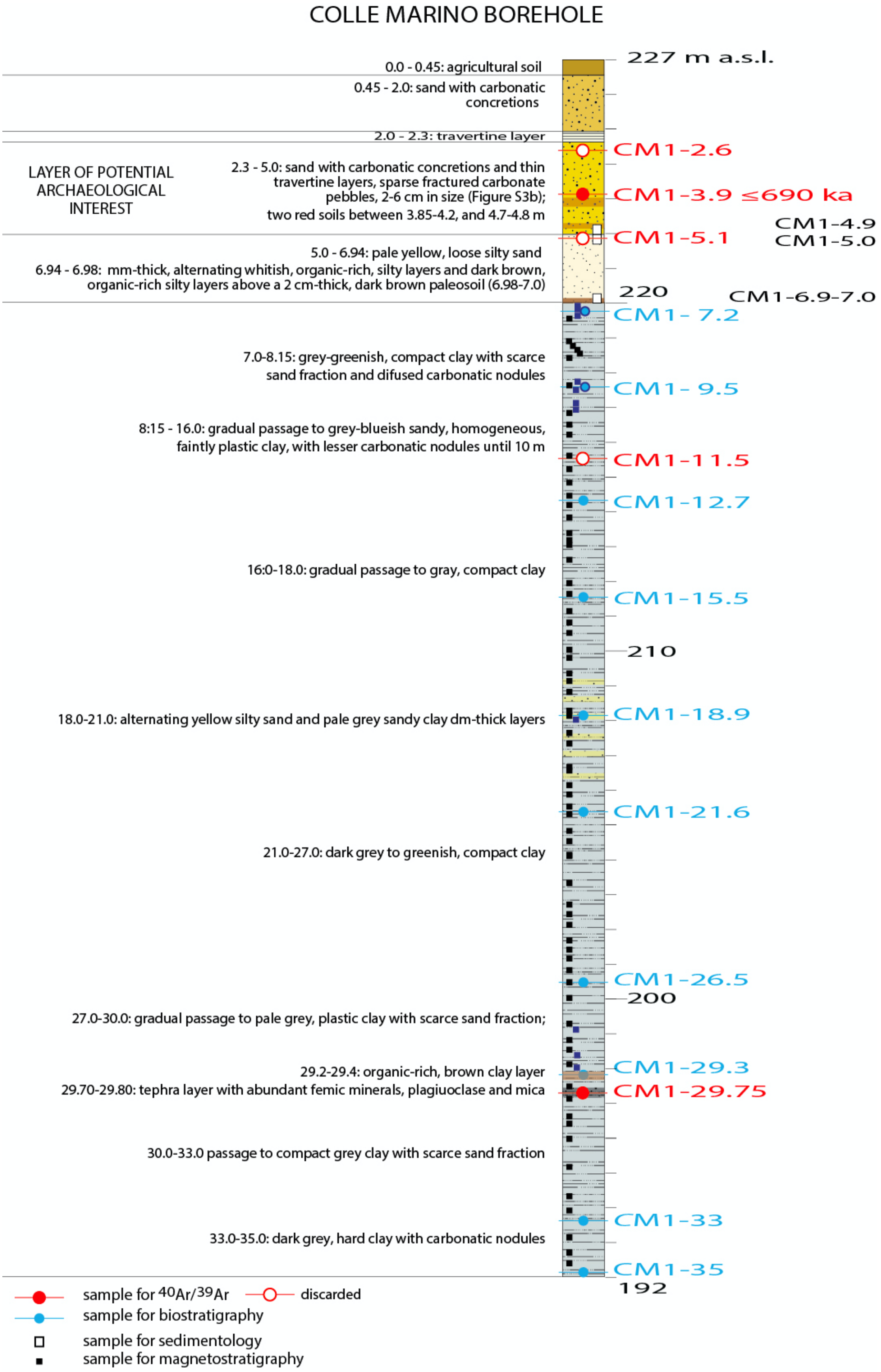

Figure S2 - detailed stratigraphic log of CM1 borehole and sampling information

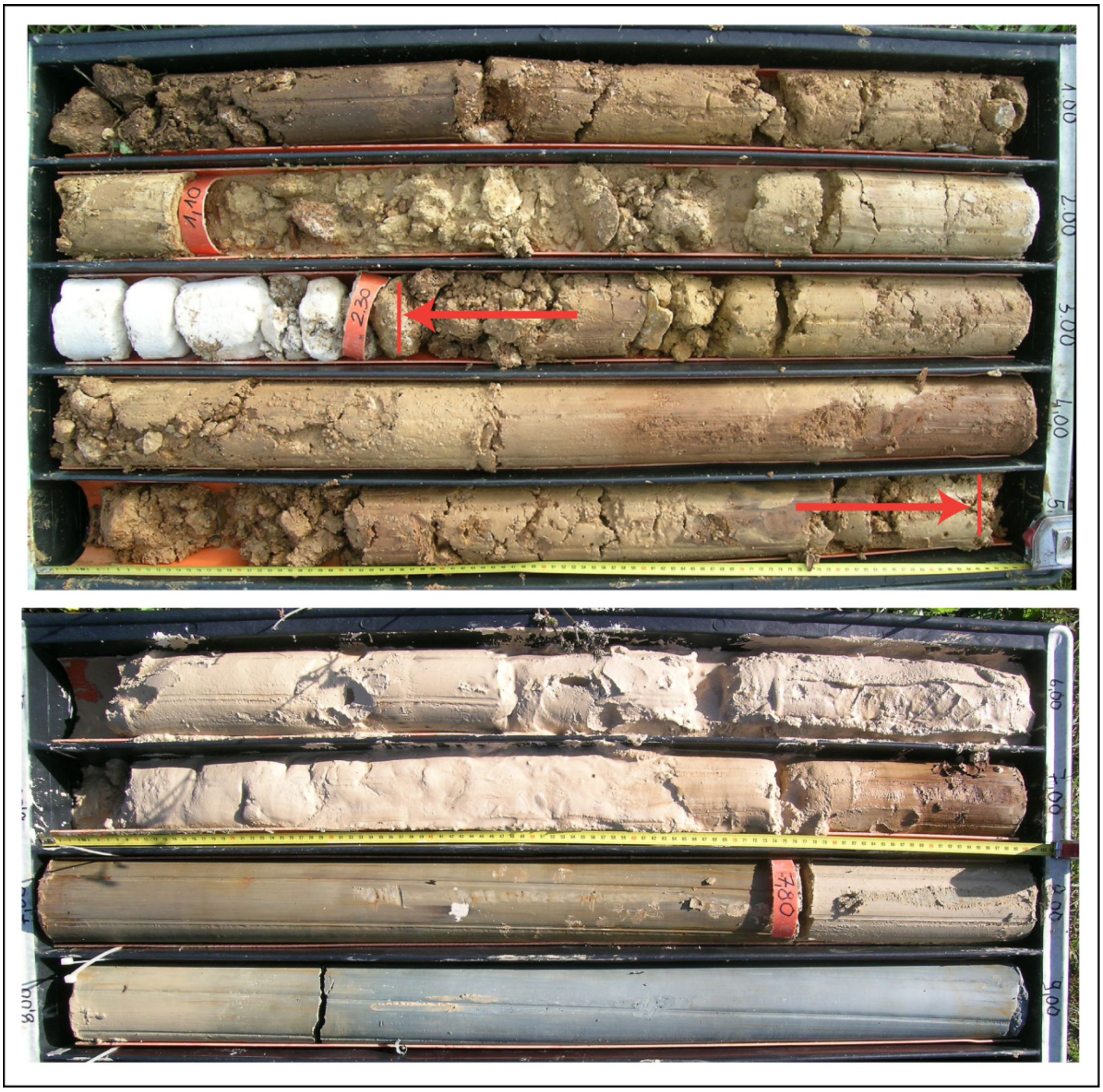

Figure S3 - a) photographs of cored sediment in the interval 0 - 9 m b.g.s.. Arrows indicate the potential archaeological layer. Pictures taken by Fabrizio Marra.

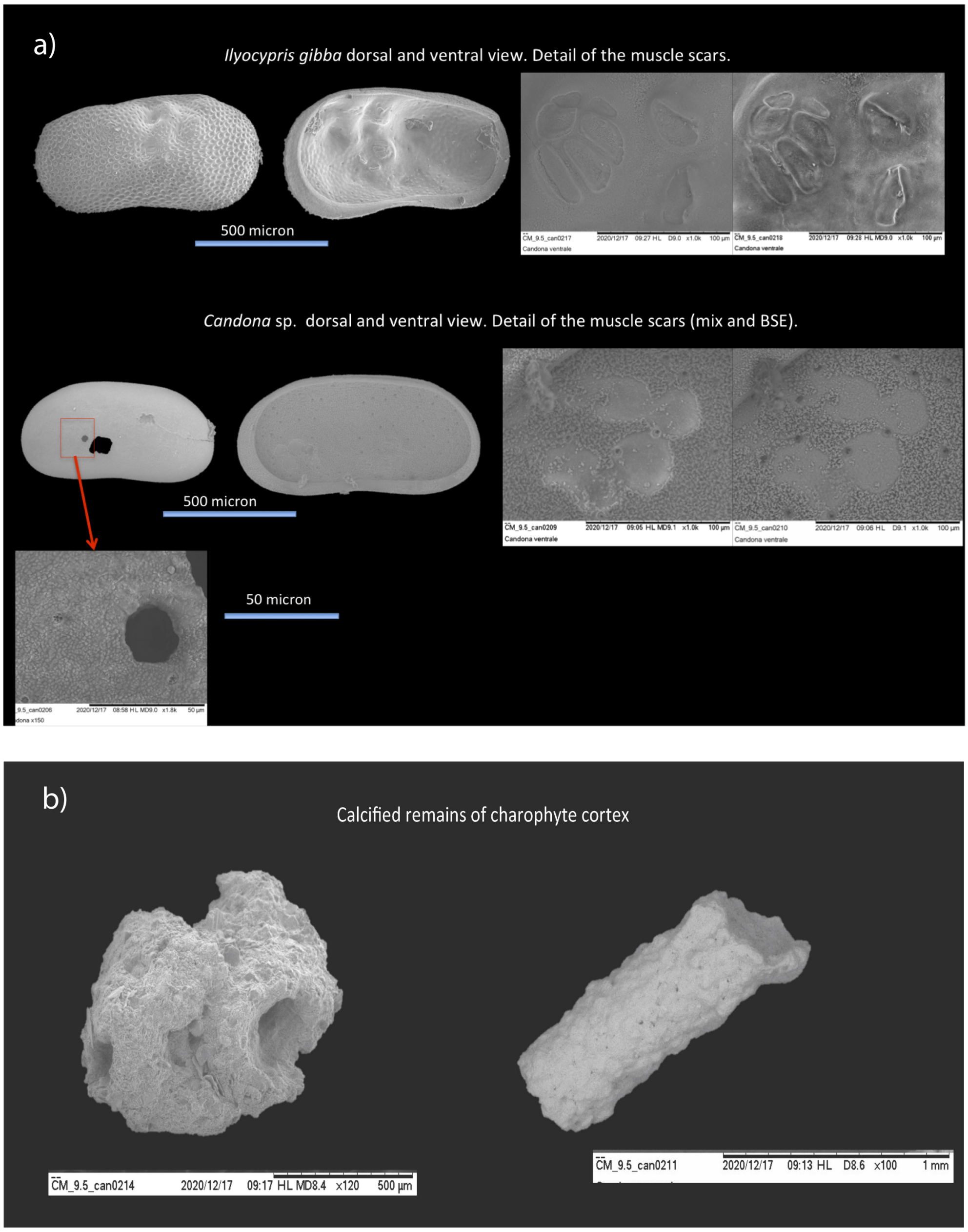

Figure S4 -Photos of ostracods (a) and calcified remains of charophyte cortex (b) occurring in sample CM 1-9.5. Pictures by Caterina Morigi.

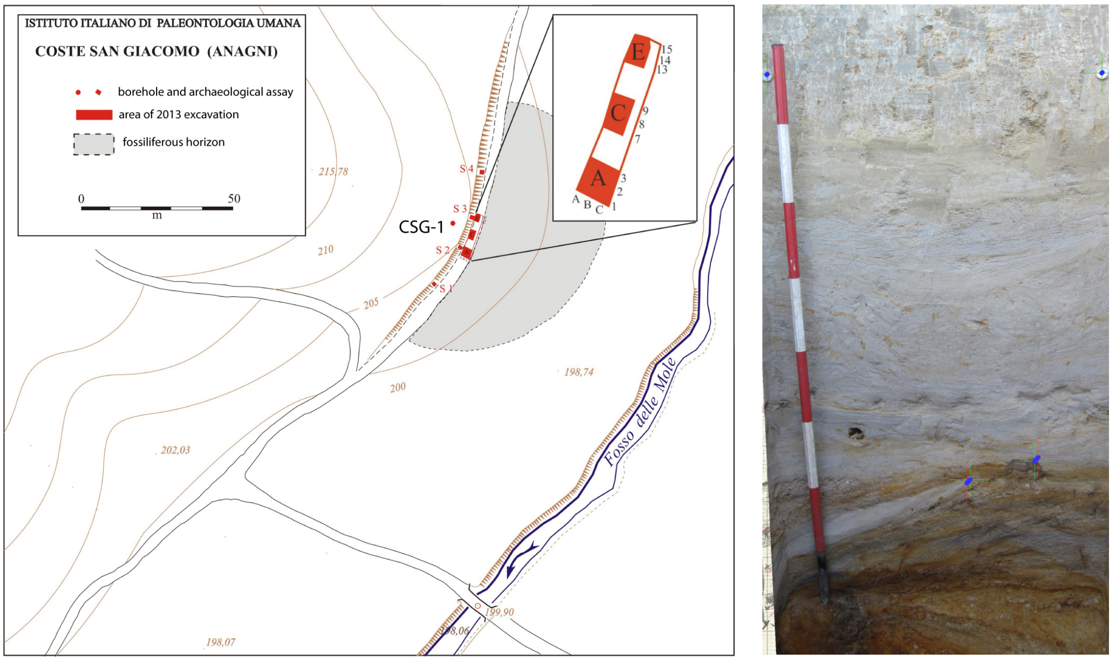

Figure S5 - a) Map showing location of Coste San Giacomo archaeological site and investigated sectors. Drawing by Fabio Parenti.

b) Photograph (by Fabio Parenti) of the trench pit 2A from where the sample CSG-13 2A was collected. Note the dark grey, femic sand at the base of the pit, possibly corresponding to a sub-primary tephra layer (see main text for comments).
