# Supplementary material for: Environmental evolution, faunal and human occupation since 2 Ma in the Anagni basin, central Italy
Source: Sci Rep. 2021 Mar 29;11:7056. doi: 10.1038/s41598-021-85446-5 (PMC8007579; doi:10.1038/s41598-021-85446-5)
Supplement: Supplementary file 2 — Supplementary Information 2. [file 41598_2021_85446_MOESM2_ESM.pdf]

Environmental evolution, faunal and human occupation since 2 Ma in the Anagni basin, central Italy

Fabio Florindo[1,2]*, Fabrizio Marra[1], Diego E. Angelucci[3], Italo Biddittu[4], Luciano Bruni[4], Federico Florindo[5], Mario Gaeta[6], Hervé Guillou[7], Brian Jicha[8], Patrizia Macrì[1], Caterina Morigi[9], Sebastien Nomade[7], Fabio Parenti[4,10], Alison Pereira[11,12], Stefano Grimaldi[3,4]
[1] Istituto Nazionale di Geofisica e Vulcanologia, Rome, Italy
[2] Institute for Climate Change Solutions, Via Sorchio snc, 61040 Frontone, Italy
[3] Dept. of Humanities, University of Trento (Trento, Italy)
[4] Istituto Italiano di Paleontologia Umana (Anagni, Italy)
[5] Sapienza Università di Roma, Piazzale Aldo Moro 5, 00185, Roma, Italy
[6] Sapienza Università di Roma, Dipartimento di Scienze della Terra, Piazzale Aldo Moro 5, 00185, Roma, Italy
[7] Laboratoire des Sciences du Climat et de l'Environnement. LSCE/IPSL, UMR CEA-CNRS-UVSQ 8212. CEA Saclay, Bat 714. Chemin de Saint Aubin - RD 128 F-91191 Gif sur Yvette France
[8] Department of Geoscience, University of Wisconsin-Madison, USA
[9] Department of Earth Sciences, University of Pisa, Via S. Maria 53, 56126 Pisa, Italy
[10] Universidade Federal do Paraná, Curitiba, Brazil
[11] Université Paris-Saclay, CNRS Laboratoire GEOPS, Orsay, France
[12] Département Hommes et environnements, Muséum national d'Histoire naturelle, Paris, France
*corresponding author: fabio.florindo@ingv.it

Supplementary Material #2A Full 40Ar/39Ar data LSCE

**Sample ID:**	**CM1-3.9**	**Lab#S0020-01-3738/S0020-21-3950**		**J = 0.00055190 ± 0.00000055**														
			**Irradiation # CO-007 (120 min)**		**reactor:**	**CICLIT, Oregon University, Triga reactor**												
**Flux standard monitor**	**ACs-2**	**1.1848 Ma**		**Single crystal total fusion**														
N	^{40}Ar	^{36}Ar	±s$_{36}$	^{37}Ar	±s$_{37}$	^{38}Ar	±s$_{38}$	^{39}Ar	±s$_{39}$	^{40}Ar	±s$_{40}$	D$^{(1)}$	±%s$_D$	%^{40}Ar*	Age	±s	K/Ca	±s
	(moles)	V	V	V	V	V	V	V	V	V	V				(ka)			
**S0020-01-3738**	1,78E-14	1,28E-06	2,72E-08	5,08E-05	7,10E-08	1,04E-04	4,11E-07	9,91E-03	3,58E-06	8,64E-03	2,97E-06	1,001	0,17	98,26	**847,1**	**± 3,6**	36,0	± 0,4
**S0020-02-3739**	1,70E-14	1,73E-06	1,33E-08	7,31E-05	1,09E-07	1,23E-04	5,69E-07	1,14E-02	4,07E-06	8,27E-03	2,61E-06	1,001	0,17	96,50	**688,8**	**± 2,7**	28,8	± 0,3
**S0020-03-3740**	7,09E-15	1,16E-06	1,85E-08	7,37E-06	5,27E-08	2,90E-05	5,29E-07	3,47E-03	1,84E-06	3,55E-03	2,03E-06	1,001	0,17	97,18	**951,0**	**± 5,7**	98,4	± 2,0
**S0020-04-3741**	1,70E-14	1,28E-06	2,62E-08	5,56E-05	1,29E-07	1,00E-04	4,30E-07	9,49E-03	4,46E-06	8,25E-03	3,41E-06	1,001	0,17	98,19	**842,7**	**± 3,7**	31,5	± 0,4
**S0020-05-3743**	3,29E-14	7,76E-06	6,06E-08	8,75E-05	2,16E-07	1,94E-04	7,58E-07	1,71E-02	1,01E-05	1,58E-02	7,97E-06	1,001	0,17	86,58	**801,5**	**± 4,2**	35,7	± 0,4
**S0020-06-3744**	2,48E-14	1,58E-06	2,44E-08	6,61E-05	1,41E-07	1,53E-04	8,18E-07	1,38E-02	7,43E-06	1,20E-02	4,99E-06	1,001	0,17	97,86	**843,6**	**± 3,4**	38,4	± 0,5
**S0020-07-3745**	1,78E-14	2,60E-06	1,39E-08	1,45E-05	6,60E-08	9,66E-05	2,50E-07	9,25E-03	3,20E-06	8,63E-03	2,72E-06	1,001	0,17	93,49	**862,5**	**± 3,5**	123,8	± 1,9
**S0020-08-3746**	8,44E-15	1,13E-06	2,57E-08	1,70E-05	7,60E-08	4,11E-05	2,45E-07	4,59E-03	2,59E-06	4,19E-03	2,01E-06	1,001	0,17	97,66	**862,3**	**± 5,2**	51,9	± 0,8
**S0020-09-3748**	2,76E-14	3,13E-06	3,16E-08	1,27E-04	1,88E-07	1,66E-04	6,72E-07	1,49E-02	4,25E-06	1,33E-02	2,44E-06	1,001	0,17	94,65	**843,8**	**± 3,4**	21,3	± 0,2
**S0020-10-3749**	6,57E-15	1,23E-06	2,12E-08	7,74E-05	2,35E-07	3,88E-05	4,26E-07	4,26E-03	2,28E-06	3,28E-03	1,27E-06	1,001	0,17	96,63	**714,3**	**± 5,2**	10,1	± 0,1
**S0020-11-3750**	9,60E-15	1,21E-06	2,34E-08	3,08E-05	5,44E-08	5,08E-05	4,11E-07	5,35E-03	2,27E-06	4,73E-03	2,73E-06	1,001	0,17	97,56	**840,7**	**± 4,9**	32,4	± 0,4
**S0020-12-3751**	6,92E-15	1,10E-06	1,69E-08	4,85E-06	4,32E-08	2,60E-05	5,21E-07	3,28E-03	2,41E-06	3,36E-03	1,20E-06	1,001	0,17	94,22	**952,0**	**± 5,4**	149,4	± 3,8
**S0020-13-3753**	8,96E-15	1,08E-06	1,72E-08	3,04E-05	1,21E-07	4,49E-05	5,50E-07	4,89E-03	3,29E-06	4,34E-03	2,52E-06	1,001	0,17	95,66	**841,7**	**± 4,2**	29,9	± 0,4
**S0020-14-3754**	8,09E-15	1,19E-06	2,08E-08	2,06E-05	7,11E-08	3,84E-05	6,27E-07	4,40E-03	1,68E-06	3,92E-03	2,11E-06	1,001	0,17	94,34	**831,8**	**± 4,6**	40,2	± 0,5
**S0020-15-3755**	3,60E-14	5,58E-06	5,13E-08	1,23E-04	1,64E-07	2,12E-04	4,18E-07	1,87E-02	8,53E-06	1,72E-02	6,29E-06	1,001	0,17	90,89	**840,3**	**± 3,7**	27,5	± 0,3
**S0020-16-3944**	6,35E-15	1,50E-06	2,18E-08	6,20E-05	1,79E-07	3,44E-05	5,79E-07	4,07E-03	2,06E-06	3,23E-03	1,36E-06	1,001	0,16	97,31	**728,5**	**± 4,7**	9,5	± 0,1
**S0020-17-3945**	1,15E-14	1,65E-06	1,02E-08	2,47E-05	7,82E-08	6,21E-05	5,96E-07	6,35E-03	1,60E-06	5,67E-03	1,54E-06	1,001	0,16	97,41	**844,6**	**± 3,3**	38,4	± 0,5
**S0020-18-3946**	2,03E-14	4,69E-06	3,51E-08	4,27E-05	1,23E-07	1,08E-04	4,14E-07	1,00E-02	3,89E-06	9,86E-03	5,41E-06	1,001	0,16	89,06	**866,9**	**± 4,1**	34,3	± 0,4
**S0020-19-3947**	5,49E-15	1,45E-06	1,36E-08	1,19E-05	7,08E-08	2,18E-05	2,44E-07	3,03E-03	1,58E-06	2,82E-03	1,06E-06	1,001	0,16	97,15	**842,9**	**± 5,0**	40,1	± 0,7
**S0020-20-3949**	5,07E-15	1,41E-06	1,05E-08	1,21E-05	6,12E-08	2,36E-05	5,72E-07	2,97E-03	1,15E-06	2,62E-03	1,16E-06	1,001	0,16	96,72	**789,8**	**± 5,7**	38,3	± 0,6
**S0020-21-3950**	9,48E-15	2,61E-06	3,17E-08	3,43E-06	2,08E-08	4,22E-05	7,56E-07	4,68E-03	2,02E-06	4,72E-03	2,08E-06	1,001	0,16	90,14	**876,3**	**± 6,0**	280,4	± 6,9

Background corrections CM1-3.9m										
N	^{36}Ar<br>V	±σ36<br>V	^{37}Ar<br>V	±σ37<br>V	^{38}Ar<br>V	±σ38<br>V	^{39}Ar<br>V	±σ39<br>V	^{40}Ar<br>V	±σ40<br>V
S0020-02-3739	8,80E-07	1,76E-08	9,66E-07	1,63E-08	1,34E-05	3,26E-07	1,37E-05	4,69E-07	1,71E-04	3,49E-07
S0020-03-3740	8,80E-07	1,76E-08	9,66E-07	1,63E-08	1,34E-05	3,26E-07	1,37E-05	4,69E-07	1,71E-04	3,49E-07
S0020-04-3741	8,80E-07	1,76E-08	9,66E-07	1,63E-08	1,34E-05	3,26E-07	1,37E-05	4,69E-07	1,71E-04	3,49E-07
S0020-05-3743	8,60E-07	1,81E-08	9,77E-07	1,69E-08	1,39E-05	4,99E-07	1,33E-05	3,49E-07	1,74E-04	7,41E-07
S0020-06-3744	8,60E-07	1,81E-08	9,77E-07	1,69E-08	1,39E-05	4,99E-07	1,33E-05	3,49E-07	1,74E-04	7,41E-07
S0020-07-3745	8,60E-07	1,81E-08	9,77E-07	1,69E-08	1,39E-05	4,99E-07	1,33E-05	3,49E-07	1,74E-04	7,41E-07
S0020-08-3746	8,60E-07	1,81E-08	9,77E-07	1,69E-08	1,39E-05	4,99E-07	1,33E-05	3,49E-07	1,74E-04	7,41E-07
S0020-09-3748	8,80E-07	2,35E-08	1,00E-06	1,67E-08	1,42E-05	3,22E-07	1,31E-05	3,59E-07	1,56E-04	6,01E-07
S0020-10-3749	8,80E-07	2,35E-08	1,00E-06	1,67E-08	1,42E-05	3,22E-07	1,31E-05	3,59E-07	1,56E-04	6,01E-07
S0020-11-3750	8,80E-07	2,35E-08	1,00E-06	1,67E-08	1,42E-05	3,22E-07	1,31E-05	3,59E-07	1,56E-04	6,01E-07
S0020-12-3751	4,98E-07	1,27E-08	8,86E-07	1,39E-08	1,41E-05	4,58E-07	1,32E-05	3,66E-07	6,64E-05	7,44E-07
S0020-13-3753	4,98E-07	1,27E-08	8,86E-07	1,39E-08	1,41E-05	4,58E-07	1,32E-05	3,66E-07	6,64E-05	7,44E-07
S0020-14-3754	4,98E-07	1,27E-08	8,86E-07	1,39E-08	1,41E-05	4,58E-07	1,32E-05	3,66E-07	6,64E-05	7,44E-07
S0020-15-3755	4,98E-07	1,27E-08	8,86E-07	1,39E-08	1,41E-05	4,58E-07	1,32E-05	3,66E-07	6,64E-05	7,44E-07
S0020-18-3946	1,23E-06	1,51E-08	1,10E-06	1,75E-08	1,54E-05	4,99E-07	1,41E-05	4,02E-07	2,08E-04	3,91E-07
S0020-21-3950	1,17E-06	2,27E-08	1,06E-06	1,62E-08	1,47E-05	4,13E-07	1,38E-05	4,24E-07	2,06E-04	6,06E-07

**Instrument: Isotopx NGX 600**				
Standard:	Alder Creek rhyolite sanidine			
Standard age (Ma):	1,1848	± 0,0006	Niespolo et al., 2017	
Atmospheric argon ratios				
$^{40}Ar/^{36}Ar$	298,56	± 0,31	Lee et al. (2006)	
$^{38}Ar/^{36}Ar$	0,1885	± 0,0003	Lee et al. (2006)	
Decay constant (Min et al., 2000)				
λ_{40Ar}	$(0.580 \pm 0.007) \times 10^{-10}\ a^{-1}$			
λ_{B^-}	$(4.884 \pm 0.0049) \times 10^{-10}\ a^{-1}$			
λ_{Tot}	$(5.463 \pm 0.0054) \times 10^{-10}\ a^{-1}$			
Nucleogenic production ratios, OSU TRIGA reactor				
$(^{36}Ar/^{37}Ar)_{Ca}$	2,65	± 0.2	$\times 10^{-4}$	
$(^{39}Ar/^{37}Ar)_{Ca}$	6,95	± 0.9	$\times 10^{-4}$	
$(^{38}Ar/^{37}Ar)_{Ca}$	0,196	± 0.00816	$\times 10^{-4}$	
$(^{40}Ar/^{39}Ar)_{K}$	7,3	± 3	$\times 10^{-4}$	
$(^{38}Ar/^{39}Ar)_{K}$	1,22	± 0.027	$\times 10^{-2}$	
$(^{36}Ar/^{38}Ar)_{Cl}$	320			
Ca/K	1,96			
Cl/K	2,9			
