# Supplementary material for: Environmental evolution, faunal and human occupation since 2 Ma in the Anagni basin, central Italy
Source: Sci Rep. 2021 Mar 29;11:7056. doi: 10.1038/s41598-021-85446-5 (PMC8007579; doi:10.1038/s41598-021-85446-5)
Supplement: Supplementary file 3 — Supplementary Information 3. [file 41598_2021_85446_MOESM3_ESM.pdf]

Environmental evolution, faunal and human occupation since 2 Ma in the Anagni basin, central Italy

Fabio Florindo[1,2*], Fabrizio Marra[1], Diego E. Angelucci[3], Italo Biddittu[4], Luciano Bruni[4], Federico Florindo[5], Mario Gaeta[6], Hervé Guillou[7], Brian Jicha[8], Patrizia Macrì[1], Caterina Morigi[9], Sebastien Nomade[7], Fabio Parenti[4,10], Alison Pereira[11,12], Stefano Grimaldi[3,4]
[1] Istituto Nazionale di Geofisica e Vulcanologia, Rome, Italy
[2] Institute for Climate Change Solutions, Via Sorchio snc, 61040 Frontone, Italy
[3] Dept. of Humanities, University of Trento (Trento, Italy)
[4] Istituto Italiano di Paleontologia Umana (Anagni, Italy)
[5] Sapienza Università di Roma, Piazzale Aldo Moro 5, 00185, Roma, Italy
[6] Sapienza Università di Roma, Dipartimento di Scienze della Terra, Piazzale Aldo Moro 5, 00185, Roma, Italy
[7] Laboratoire des Sciences du Climat et de l'Environnement. LSCE/IPSL, UMR CEA-CNRS-UVSQ 8212. CEA Saclay, Bat 714. Chemin de Saint Aubin - RD 128 F-91191 Gif sur Yvette France
[8] Department of Geoscience, University of Wisconsin-Madison, USA
[9] Department of Earth Sciences, University of Pisa, Via S. Maria 53, 56126 Pisa, Italy
[10] Universidade Federal do Paraná, Curitiba, Brazil
[11] Université Paris-Saclay, CNRS Laboratoire GEOPS, Orsay, France
[12] Département Hommes et environnements, Muséum national d'Histoire naturelle, Paris, France
*corresponding author: fabio.florindo@ingv.it

Supplementary Material #2B Full 40Ar/39Ar data WISCAR

	**Sample:**	**CM1**			**Identifier:**	**UW152:B51**									
	**Material:**	**Sanidine**													
														Corrected[1]	
	N	Power	Age	±1σ	K/Ca	±1σ	%^{40}Ar	^{40}Ar*/^{39}Ar$_K$	±1σ	^{39}Ar/^{40}Ar	±1σ	^{36}Ar/^{40}Ar	±1σ	^{40}Ar	±1σ
		%	(Ma)	(Ma)			(%)							(cps)	
	01A	18	2,185	0,106	0,0502	0,0004	34,945291	0,7116021	0,0344236	0,4924	0,0008	0,00218	0,00006	35568,40026	44,26334853
	02A	18	2,093	0,098	0,0455	0,0003	22,146553	0,6816943	0,0317951	0,3254	0,0004	0,00261	0,00003	92320,46572	72,32336594
	04A	18	2,203	0,122	0,0512	0,0004	24,491237	0,7174002	0,0397195	0,3420	0,0006	0,00253	0,00005	50756,57035	62,17716601
	07A	18	2,249	0,053	0,0549	0,0004	43,067480	0,7326549	0,0173383	0,5897	0,0005	0,00190	0,00003	113470,8355	80,16008459
	08A	18	2,285	0,082	0,0518	0,0004	45,993974	0,7442974	0,0268342	0,6200	0,0009	0,00180	0,00006	34628,61379	42,04851962
	09A	18	2,158	0,071	0,0487	0,0004	36,609590	0,7029479	0,0232488	0,5223	0,0005	0,00212	0,00004	72352,29332	55,55233618
	10A	18	2,064	0,086	0,0491	0,0004	23,107669	0,6722867	0,0279957	0,3444	0,0003	0,00257	0,00003	61868,51257	45,46023275
	11A	18	2,211	0,077	0,0487	0,0004	21,493053	0,7200715	0,0251824	0,2990	0,0003	0,00263	0,00003	99033,88111	63,11151907
	12A	18	2,264	0,082	0,0479	0,0004	23,558578	0,7375755	0,0268087	0,3200	0,0003	0,00256	0,00003	68308,2239	46,50746751
	13A	18	2,281	0,056	0,0509	0,0004	38,206603	0,7428741	0,0183107	0,5157	0,0004	0,00207	0,00003	70979,88081	47,2652922
	14A	18	2,097	0,066	0,0505	0,0004	18,777551	0,6830696	0,0214764	0,2753	0,0002	0,00272	0,00002	203717,4755	123,7626739
	15A	18	2,094	0,065	0,0509	0,0004	18,859090	0,6821583	0,0211671	0,2769	0,0002	0,00272	0,00002	259184,2983	148,942786
	16A	18	2,244	0,074	0,0459	0,0003	27,739532	0,7310605	0,0239626	0,3802	0,0003	0,00242	0,00003	71574,6906	49,14747892
	17A	18	2,230	0,125	0,0366	0,0003	16,476514	0,7264092	0,0408537	0,2271	0,0003	0,00280	0,00003	69603,53835	54,46679633
	18A	18	2,246	0,058	0,0451	0,0003	36,239797	0,7314819	0,0189441	0,4968	0,0004	0,00213	0,00003	93781,57234	59,4854388
	19A	18	2,142	0,067	0,0469	0,0003	25,356679	0,6978288	0,0219654	0,3641	0,0003	0,00250	0,00003	99396,42036	64,89482645
	20A	18	2,274	0,048	0,0563	0,0004	52,136337	0,7405999	0,0157848	0,7067	0,0006	0,00160	0,00004	55861,64622	37,18632763
	21A	18	2,299	0,067	0,0467	0,0003	29,272541	0,7489676	0,0219168	0,3917	0,0003	0,00237	0,00003	86662,11914	56,60699185
	22A	18	2,262	0,084	0,0362	0,0003	38,511704	0,7366245	0,0275097	0,5243	0,0006	0,00206	0,00005	36436,33248	31,10635362
**Weighted mean K/Ca ±2σ**					**0,046846**	**0,002619**									
**Weighted mean Age ±2σ**			**2,214**	**0,034**	**n=19/22**	**MSWD=1.103**									

^{36}Ar	±1σ	RunDate	Δt[3]	J	±1σ	^{39}Ar Decay	^{37}Ar Decay	LambdaK	MonitorAge	MonitorName	MonitorMaterial	$(^{40}Ar/^{39}39)_K$
(cps)			(days)									
116,9207062	1,890813082	08/11/20 16:16	173,0938079	0,0016785	0,0000009	1,001221	30,259479	5,464E-10	1,1864	ACS-2	sanidine	0,005400
315,5392104	2,912516609	08/11/20 16:54	173,1202778	0,0016785	0,0000009	1,001221	30,275302	5,464E-10	1,1864	ACS-2	sanidine	0,005400
166,6949815	2,196536242	08/11/20 17:55	173,1627199	0,0016785	0,0000009	1,001222	30,300690	5,464E-10	1,1864	ACS-2	sanidine	0,005400
353,8860341	3,075513037	08/11/20 19:28	173,2271065	0,0016785	0,0000009	1,001222	30,339246	5,464E-10	1,1864	ACS-2	sanidine	0,005400
109,404499	1,741203461	08/11/20 20:15	173,2598611	0,0016785	0,0000009	1,001222	30,358879	5,464E-10	1,1864	ACS-2	sanidine	0,005400
241,3201978	2,513241247	08/11/20 20:37	173,2756829	0,0016785	0,0000009	1,001222	30,368367	5,464E-10	1,1864	ACS-2	sanidine	0,005400
208,4215738	1,796088848	08/11/20 21:07	173,2964468	0,0016785	0,0000009	1,001222	30,380823	5,464E-10	1,1864	ACS-2	sanidine	0,005400
329,1466952	2,1784477	08/11/20 21:55	173,3294097	0,0016785	0,0000009	1,001223	30,400608	5,464E-10	1,1864	ACS-2	sanidine	0,005400
226,4877652	1,736796497	08/11/20 22:16	173,3440278	0,0016785	0,0000009	1,001223	30,409386	5,464E-10	1,1864	ACS-2	sanidine	0,005400
228,2322229	1,752560268	08/11/20 22:39	173,3600579	0,0016785	0,0000009	1,001223	30,419015	5,464E-10	1,1864	ACS-2	sanidine	0,005400
679,9703962	3,351707196	08/11/20 23:26	173,3929745	0,0016785	0,0000009	1,001223	30,438797	5,464E-10	1,1864	ACS-2	sanidine	0,005400
863,9448417	4,219240202	08/11/20 23:49	173,4089699	0,0016785	0,0000009	1,001223	30,448415	5,464E-10	1,1864	ACS-2	sanidine	0,005400
240,3532242	1,846641028	08/12/20 00:12	173,4249653	0,0016785	0,0000009	1,001223	30,458035	5,464E-10	1,1864	ACS-2	sanidine	0,005400
243,7567151	1,976011831	08/12/20 01:00	173,4578704	0,0016785	0,0000009	1,001224	30,477836	5,464E-10	1,1864	ACS-2	sanidine	0,005400
316,9996946	2,174276371	08/12/20 01:21	173,4724884	0,0016785	0,0000009	1,001224	30,486636	5,464E-10	1,1864	ACS-2	sanidine	0,005400
335,7702933	2,184908178	08/12/20 01:42	173,4871528	0,0016785	0,0000009	1,001224	30,495467	5,464E-10	1,1864	ACS-2	sanidine	0,005400
168,6807518	1,583937724	08/12/20 02:27	173,5187384	0,0016785	0,0000009	1,001224	30,514496	5,464E-10	1,1864	ACS-2	sanidine	0,005400
287,5473603	2,042955071	08/12/20 02:49	173,5333449	0,0016785	0,0000009	1,001224	30,523300	5,464E-10	1,1864	ACS-2	sanidine	0,005400
134,8506886	1,430401432	08/12/20 03:10	173,5479977	0,0016785	0,0000009	1,001224	30,532135	5,464E-10	1,1864	ACS-2	sanidine	0,005400

	**Sample:**	**CSG-13**			**Identifier:**	**UW152:B50**									
	**Material:**	**Sanidine**													
														Corrected[1]	
	N	Power	Age	±1σ	K/Ca	±1σ	%^{40}Ar	^{40}Ar*/^{39}Ar$_K$	±1σ	^{39}Ar/^{40}Ar	±1σ	^{36}Ar/^{40}Ar	±1σ	^{40}Ar	±1σ
		%	(Ma)	(Ma)			(%)							(cps)	
	01A	18	2,220	0,076	0,0458	0,0003	28,607729	0,7231566	0,0249130	0,3964	0,0004	0,00239	0,00003	102989,4186	79,16125016
X	02A	18	2,574	0,068	0,0475	0,0003	47,791172	0,8385767	0,0221088	0,5717	0,0007	0,00174	0,00004	58533,65553	51,76698889
	03A	18	2,262	0,064	0,0487	0,0003	57,369658	0,7368501	0,0208994	0,7819	0,0007	0,00142	0,00005	85115,73126	65,53120229
	04A	18	2,371	0,060	0,0456	0,0003	76,791515	0,7723830	0,0194654	1,000	0,001	0,00076	0,00007	83291,69746	67,92489066
	05A	18	2,268	0,051	0,0471	0,0003	51,712931	0,7387049	0,0167420	0,7027	0,0007	0,00161	0,00004	121643,5071	92,285606
	06A	18	2,236	0,056	0,0438	0,0003	69,425584	0,7283479	0,0182546	0,9581	0,0010	0,00101	0,00006	96846,93193	78,61223936
	07A	18	2,129	0,067	0,0443	0,0003	25,826663	0,6932813	0,0219896	0,3733	0,0003	0,00248	0,00003	245616,9362	149,5141033
	08A	18	2,272	0,054	0,0472	0,0003	43,409769	0,7401371	0,0177140	0,5884	0,0005	0,00189	0,00003	129840,8867	87,68798973
	09A	18	2,336	0,056	0,0481	0,0004	73,913842	0,7609381	0,0182274	0,9765	0,0009	0,00086	0,00006	85212,81759	61,17038633
	10A	18	2,231	0,046	0,0519	0,0004	53,194902	0,7266737	0,0149843	0,7349	0,0006	0,00156	0,00004	121546,929	85,1060713
	13A	18	2,273	0,059	0,0441	0,0003	66,432005	0,7405101	0,0190794	0,901	0,001	0,00111	0,00006	49839,41035	46,64806354
X	14A	18	2,467	0,067	0,0411	0,0003	46,537012	0,8036713	0,0219164	0,5809	0,0005	0,00179	0,00004	157131,9554	95,69119006
	18A	18	2,102	0,063	0,0523	0,0005	38,078695	0,6846866	0,0206325	0,5578	0,0006	0,00207	0,00004	98694,21218	85,16601221
	21A	18	2,200	0,070	0,0453	0,0003	52,739643	0,7166998	0,0228388	0,739	0,001	0,00158	0,00006	42513,62124	45,94736616
	22A	18	2,153	0,056	0,0491	0,0004	37,096259	0,7011027	0,0183751	0,5306	0,0004	0,00210	0,00003	146998,7794	97,15889089
X	23A	18	2,602	0,064	0,0439	0,0003	42,604212	0,8476971	0,0209391	0,5040	0,0005	0,00192	0,00004	107769,0718	77,61612271
	25A	18	2,210	0,048	0,0492	0,0004	53,706075	0,7199763	0,0155564	0,7490	0,0006	0,00154	0,00004	128621,8912	88,55449963
	26A	18	2,131	0,111	0,0405	0,0003	18,426599	0,6940716	0,0360766	0,2659	0,0003	0,00273	0,00003	116081,0452	111,0231147
	27A	18	2,216	0,058	0,0474	0,0003	71,824450	0,7219372	0,0189282	1,0003	0,0010	0,00093	0,00006	72441,58967	57,55448635
	28A	18	2,261	0,060	0,0465	0,0003	72,296324	0,7366041	0,0195267	0,987	0,001	0,00092	0,00006	61627,18632	49,41973444
X	29A	18	4,236	0,125	0,0427	0,0003	29,463541	1,3805366	0,0406930	0,2137	0,0003	0,00236	0,00003	95652,60163	69,02349821
	31A	18	2,152	0,091	0,0467	0,0003	17,982137	0,7009540	0,0295644	0,2569	0,0002	0,00275	0,00003	210070,3944	130,8501046
**Weighted mean K/Ca ±2σ**					**0,046400**	**0,001303**									
**Weighted mean Age ±2σ**			**2,233**	**0,032**	**n=18/22**	**MSWD=1.268**									
**Notes:**															
[1]Corrected: Isotopic intensities corrected for blank, baseline, radioactive decay and detector intercalibration, not for interfering reactions.															
[3]Time interval (days) between end of irradiation and beginning of analysis.															
X symbol preceding sample ID denotes analyses excluded from weighted-mean age calculations.															
Errors quoted for individual analyses include analytical error only, without interfering reaction or J uncertainties.															
Decay constants and isotopic abundances after Min et al. (2000)															
Ages calculated relative to 1.1864 Ma Alder Creek sanidine standard (Jicha et al., 2016)															

^{36}Ar	±1σ	RunDate	Δt[3]	J	±1σ	^{39}Ar Decay	^{37}Ar Decay	LambdaK	MonitorAge	MonitorName	MonitorMaterial	$(^{40}Ar/^{39}39)_K$
(cps)			(days)									
347,2271581	2,918255502	08/11/20 02:38	172,5263657	0,0016785	0,0000009	1,001217	29,922255	5,464E-10	1,1864	ACS-2	sanidine	0,005400
181,9358731	2,060841626	08/11/20 02:52	172,5359606	0,0016785	0,0000009	1,001217	29,927925	5,464E-10	1,1864	ACS-2	sanidine	0,005400
275,7616351	3,842075342	08/11/20 03:30	172,562419	0,0016785	0,0000009	1,001217	29,943568	5,464E-10	1,1864	ACS-2	sanidine	0,005400
270,7096426	4,143713017	08/11/20 03:44	172,5719907	0,0016785	0,0000009	1,001217	29,949229	5,464E-10	1,1864	ACS-2	sanidine	0,005400
401,721947	3,294939393	08/11/20 03:58	172,5815741	0,0016785	0,0000009	1,001217	29,954898	5,464E-10	1,1864	ACS-2	sanidine	0,005400
338,267975	3,95746808	08/11/20 04:36	172,6079861	0,0016785	0,0000009	1,001218	29,970528	5,464E-10	1,1864	ACS-2	sanidine	0,005400
844,5275407	5,412179109	08/11/20 04:50	172,6176157	0,0016785	0,0000009	1,001218	29,976229	5,464E-10	1,1864	ACS-2	sanidine	0,005400
428,8749706	3,290331501	08/11/20 05:04	172,6272106	0,0016785	0,0000009	1,001218	29,981910	5,464E-10	1,1864	ACS-2	sanidine	0,005400
269,5476596	3,819548386	08/11/20 05:42	172,6537269	0,0016785	0,0000009	1,001218	29,997615	5,464E-10	1,1864	ACS-2	sanidine	0,005400
384,8970682	2,997800517	08/11/20 05:56	172,663287	0,0016785	0,0000009	1,001218	30,003280	5,464E-10	1,1864	ACS-2	sanidine	0,005400
171,1131673	2,078698176	08/11/20 07:01	172,7089815	0,0016785	0,0000009	1,001218	30,030369	5,464E-10	1,1864	ACS-2	sanidine	0,005400
532,5960441	5,130550708	08/11/20 07:15	172,7185532	0,0016785	0,0000009	1,001218	30,036046	5,464E-10	1,1864	ACS-2	sanidine	0,005400
323,6134354	3,073996144	08/11/20 09:03	172,7936111	0,0016785	0,0000009	1,001219	30,080605	5,464E-10	1,1864	ACS-2	sanidine	0,005400
145,7021051	1,986467974	08/11/20 10:17	172,8446991	0,0016785	0,0000009	1,001219	30,110971	5,464E-10	1,1864	ACS-2	sanidine	0,005400
489,3220732	3,682047806	08/11/20 10:32	172,8550347	0,0016785	0,0000009	1,001219	30,117118	5,464E-10	1,1864	ACS-2	sanidine	0,005400
347,1306713	2,959433366	08/11/20 10:47	172,8653704	0,0016785	0,0000009	1,001219	30,123266	5,464E-10	1,1864	ACS-2	sanidine	0,005400
420,3032663	3,321046295	08/11/20 11:41	172,9030903	0,0016785	0,0000009	1,001220	30,145716	5,464E-10	1,1864	ACS-2	sanidine	0,005400
403,6252679	3,390042522	08/11/20 11:56	172,9134491	0,0016785	0,0000009	1,001220	30,151884	5,464E-10	1,1864	ACS-2	sanidine	0,005400
240,7137282	3,520216523	08/11/20 12:35	172,9406713	0,0016785	0,0000009	1,001220	30,168099	5,464E-10	1,1864	ACS-2	sanidine	0,005400
204,554028	3,072345359	08/11/20 12:50	172,9509491	0,0016785	0,0000009	1,001220	30,174223	5,464E-10	1,1864	ACS-2	sanidine	0,005400
280,2565127	2,604304096	08/11/20 13:05	172,9612153	0,0016785	0,0000009	1,001220	30,180342	5,464E-10	1,1864	ACS-2	sanidine	0,005400
707,9708307	4,800190454	08/11/20 16:01	173,0835301	0,0016785	0,0000009	1,001221	30,253337	5,464E-10	1,1864	ACS-2	sanidine	0,005400
