# Supplementary material for: Environmental evolution, faunal and human occupation since 2 Ma in the Anagni basin, central Italy
Source: Sci Rep. 2021 Mar 29;11:7056. doi: 10.1038/s41598-021-85446-5 (PMC8007579; doi:10.1038/s41598-021-85446-5)
Supplement: Supplementary file 4 — Supplementary Information 4. [file 41598_2021_85446_MOESM4_ESM.pdf]

Environmental evolution, faunal and human occupation since 2 Ma in the Anagni basin, central Italy

Fabio Florindo1,2*, Fabrizio Marra1, Diego E. Angelucci3, Italo Biddittu4, Luciano Bruni4, Federico Florindo5, Mario Gaeta6, Hervé Guillou7, Brian Jicha8, Patrizia Macrì1, Caterina Morigi9, Sebastien Nomade7, Fabio Parenti4,10, Alison Pereira11,12, Stefano Grimaldi3,4

1 Istituto Nazionale di Geofisica e Vulcanologia, Rome, Italy
2 Institute for Climate Change Solutions, Via Sorchio snc, 61040 Frontone, Italy
3 Dept. of Humanities, University of Trento (Trento, Italy)
4 Istituto Italiano di Paleontologia Umana (Anagni, Italy)
5 Sapienza Università di Roma, Piazzale Aldo Moro 5, 00185, Roma, Italy
6 Sapienza Università di Roma, Dipartimento di Scienze della Terra, Piazzale Aldo Moro 5, 00185, Roma, Italy
7 Laboratoire des Sciences du Climat et de l'Environnement. LSCE/IPSL, UMR CEA-CNRS-UVSQ 8212. CEA Saclay, Bat 714. Chemin de Saint Aubin - RD 128 F-91191 Gif sur Yvette France
8 Department of Geoscience, University of Wisconsin-Madison, USA
9 Department of Earth Sciences, University of Pisa, Via S. Maria 53, 56126 Pisa, Italy
10 Universidade Federal do Paraná, Curitiba, Brazil
11 Université Paris-Saclay, CNRS Laboratoire GEOPS, Orsay, France
12 Département Hommes et environnements, Muséum national d'Histoire naturelle, Paris, France

Supplementary Material #3 Paleomagnetism

Sample code	depth	susceptibility (SI)	intensità ARM (A/m)	NRM intensity (A/m)	decl NRM (°)	Incl NRM (°)	decl ChRM (°)	Incl ChRM (°)	MAD (°)
3H	7.08	1.03E-04	1.70E-03	1.26E-04	282	56.5	345.79	51.79	13.91
3G	7.11	1.19E-04	1.31E-03	3.31E-04	1.5	48.7	345.98	45.26	5.3
3F	7.22	1.34E-04	1.50E-03	3.99E-04	346.5	27.5	350.05	21.51	6.95
CM1	7.48	8.22E-05	2.58E-03	5.98E-04	221.1	54.7	225.72	57.08	1.63
CM2d	8.13	1.03E-04	1.61E-03	1.78E-04	27	61.3	54.27	42.35	8.34
CM2c	8.35	1.13E-04	1.60E-03	9.24E-05	30.5	21.9			
CM2b	8.46	1.22E-04	1.72E-03	7.69E-05	43.9	-20.8			
CM2a	8.54	1.19E-04	1.77E-03	4.26E-05	143.1	-1.5			
CM2	8.6	1.16E-04	2.88E-03	2.08E-04	347.6	6.4			
3D	9.18	1.37E-04	1.91E-03	3.89E-05	158.4	62.1	70.57	-21.01	5.12
CM3	9.41	1.36E-04	4.03E-03	4.13E-04	163.9	-50.3	148.33	-50.59	11.22
3E	9.52	1.37E-04	2.71E-03	3.13E-04	262.7	85.6	266.85	63.17	2.96
3C	9.96	1.38E-04	2.41E-03	6.95E-04	33.7	-3.2	33.65	6.85	2.55
3B	10.11	1.14E-04	2.03E-03	4.52E-04	253.9	54.2	209.01	25.19	14.51
CM4	10.18	1.31E-04	3.26E-03	4.14E-04	113.4	21.5	104.31	10.28	12.12
CM5	10.51	1.39E-04	2.46E-03	5.56E-04	90.2	-39.5	110.24	-55.72	16.64
CM6	10.8	1.69E-04	3.17E-03	1.31E-03	326.8	-79.2	25.97	-68.72	5.89
CM7	11.42	2.47E-04	8.21E-03	2.60E-03	51.9	-50.1	22.34	-60.32	6.96
CM8	11.79	1.46E-04	4.47E-03	1.09E-03	321.1	-83.3	146.41	-76.96	1.96
CM9	12.17	1.38E-04	5.75E-03	1.14E-03	114.5	-48.6	114.04	-44.48	4.44
CM10	12.55	1.56E-04	5.03E-03	7.06E-04	46.1	-43.1	26.1	-31.6	7.88
CM11	12.83	1.40E-04	5.51E-03	9.85E-04	167.5	-59.1	183.87	-63.56	5.44
CM12	13.19	2.34E-04	5.88E-03	1.77E-03	56.9	-53.8	156.04	-27.22	3.28
CM13	13.63	2.44E-04	6.83E-03	2.11E-03	79.3	-62.8	90.6	-70.41	3.9
CM14	13.8	2.25E-04	6.27E-03	2.06E-03	109.9	-56.4	81.16	-53.16	7.86
CM15	13.97	2.48E-04	6.84E-03	1.56E-03	145.3	-67.2	113.42	-57.67	5.99
CM16	14.4	2.22E-04	6.15E-03	1.71E-03	19.7	-72.5	354.82	-53.68	10.17
CM17	15.07	2.36E-04	7.12E-03	9.70E-04	90.7	-50.9	146.27	-49.98	3.59
CM18	15.35	2.14E-04	6.35E-03	2.08E-03	79	-86.2	188.99	-69.4	7.42
CM19	15.85	2.49E-04	8.75E-03	4.36E-03	122.7	-69.7	128.54	-69.56	5.67
CM20	16.18	1.36E-04	4.48E-03	9.14E-04	357.8	-48	244.55	-23.25	7.66
CM21	16.5	1.24E-04	3.62E-03	1.46E-03	110.1	-51.4	109.74	-53.45	1.01
CM22	16.97	1.31E-04	2.51E-03	2.75E-04	79.6	-29.4			
CM23	17.2	1.41E-04	2.44E-03	8.21E-04	125.9	-60.2	119.89	-62.97	3.29
CM24	17.63	1.46E-04	3.02E-03	8.80E-04	201.6	-87.1	199.79	-45.46	3.77
CM25	17.86	1.77E-04	4.36E-03	7.37E-04	119.7	-44.8			
CM26	18.18	1.61E-04	4.46E-03	1.19E-03	2.4	-72.7	11.74	-68.43	5.26
CM27	18.71	3.01E-04	2.20E-02	2.66E-03	192.6	-61.4	187.39	-61.61	8.93
CM28	18.86	2.72E-04	4.32E-02	3.76E-03	88	-74.4	65.89	-60.61	1.84
28B	18.97	2.99E-04	2.71E-02	3.81E-03	351	-63.1	328.15	-65.2	5.04
CM29	19.35	3.40E-04	3.19E-02	4.31E-03	270.8	67.9	175.97	71.11	9.83
CM30	19.65	3.48E-04	3.49E-02	3.37E-03	286.8	76.3	249.03	67.16	8.45
CM31	20.32	2.05E-04	5.46E-03	1.31E-03	20.6	-31.7	43.68	-31.53	2.68
CM32	20.43	1.55E-04	8.29E-03	1.08E-03	103.2	-76.1			
CM33	20.85	1.58E-04	6.29E-03	2.91E-04	297.8	-34.1			
CM34	21.12	3.26E-04	1.64E-02	1.12E-03	75.6	-22.4	68.05	-61.99	11.24
CM35	21.47	2.37E-04	2.96E-02	5.42E-04	199.3	32.6			
CM36	21.66	2.75E-04	3.06E-02	8.76E-04	281.9	-66.2	219.44	-57.73	8.88
CM37	22.15	1.77E-04	7.80E-03	1.26E-03	98	-21.8			
CM38	22.46	1.28E-04	4.63E-03	1.42E-03	18.8	-9.5			
CM39	22.84	1.13E-04	3.77E-03	6.63E-04	121.5	-65	66.79	-55.73	7.48
CM40	22.88	1.19E-04	3.12E-03	7.88E-04	34.3	-77.9	48.69	-44.85	14.61
CM41	23.65	1.35E-04	3.44E-03	7.26E-04	32.2	-82.5			
CM42	24.28	2.04E-04	4.69E-03	1.75E-03	62	-45.1	24.76	-60.84	4.07
CM43	24.53	1.79E-04	4.39E-03	9.93E-04	1.6	-63.4	182.26	-59.53	7.14
CM44	24.84	1.42E-04	3.40E-03	6.97E-04	92.4	-68.8	202.39	-63.09	8.2
CM45	25.3	2.03E-04	5.48E-03	1.13E-03	78.9	-72.9	104.5	-60.64	16.05
CM46	25.57	2.18E-04	5.98E-03	9.44E-04	71.2	-44.1			
CM47	25.8	2.24E-04	5.97E-03	1.96E-03	51.9	-65.6			
CM48	26.17	1.35E-04	3.57E-03	5.97E-04	13.2	-79.6	84.33	-74.24	17.13
CM49	26.5	1.47E-04	4.71E-03	4.43E-04	128.9	-43.8			
CM50	26.96	1.80E-04	5.60E-03	1.45E-03	12.2	-61.5	35.74	-51.82	12.01
CM51	27.7	1.44E-04	6.32E-03	7.81E-04	341	-84.2	338.05	-26.3	9.83
51B	27.8	2.17E-04	1.55E-02	1.30E-03	60	-49.7	55.12	-31.6	14.03
CM52	28.17	1.18E-04	4.67E-03	1.81E-04	217.1	-65.9			
CM53	28.45	1.01E-04	5.33E-03	4.27E-04	151.1	62.9	218.07	74.06	10.32
53B	28.72	1.02E-04	2.34E-03	2.53E-04	148.8	32.2	100.91	51.42	13.44
CM54	28.85	8.66E-05	3.80E-03	6.35E-04	144.2	-39.7	152.1	-32.52	5.68
54B	28.93	1.21E-04	5.68E-03	5.92E-04	120.6	58.5			
CM55	29.5	1.17E-02	1.70E-01	5.12E-02	316.4	-54.8	303.01	-35.9	3.32
CM56	29.85	8.53E-05	4.22E-03	1.18E-03	135.5	-23.3	101.79	-31.05	20.56
CM57	30.35	1.87E-04	7.72E-03	1.62E-03	75.6	-82.6	240.95	-62.97	5.29
CM58	30.56	2.13E-04	1.31E-02	2.48E-03	61.6	-50.8	66.06	-61.1	9.73
CM59	31	1.94E-04	8.60E-03	3.05E-03	70	-47.1	80.27	-41.52	2.33
CM60	31.8	3.69E-04	8.66E-03	2.25E-03	61.1	-41.2			
CM61	32.64	2.01E-04	6.88E-03	1.54E-03	8.3	-72	338.05	-47.56	4.78
CM62	33.05	2.12E-04	9.28E-03	2.32E-03	83.7	-71.3	133.66	-48.39	8.38
CM63	33.8	1.78E-04	5.30E-03	1.74E-03	33.1	-61.5	59.5	-42.24	12.4
CM64	34.22	2.49E-04	7.19E-03	1.68E-03	64.7	-62.9	62.78	-62.09	15.01
CM65	34.55	1.41E-04	4.04E-03	8.38E-04	144	-39	164.1	-48.58	6.1
